# Elucidating the Oligomerization and Cellular Interactions of a Trimer Derived from Aβ through Fluorescence and Mass Spectrometric Studies

**DOI:** 10.1021/acschemneuro.2c00313

**Published:** 2022-07-27

**Authors:** Gretchen Guaglianone, Belén Torrado, Yu-Fu Lin, Matthew C. Watkins, Vicki H. Wysocki, Enrico Gratton, James S. Nowick

**Affiliations:** † Department of Chemistry, University of California, Irvine, Irvine, California 92697, United States; ‡ Department of Pharmaceutical Sciences, University of California, Irvine, Irvine, California 92697, United States; § Laboratory for Fluorescence Dynamics, Biomedical Engineering, University of California, Irvine, California 92697, United States; ∥ Resource for Native MS Guided Structural Biology, The Ohio State University, Columbus, Ohio 43210, United States; ⊥ Department of Chemistry and Biochemistry, The Ohio State University, Columbus, Ohio 43210, United States

**Keywords:** Alzheimer’s disease, amyloid β, oligomer, FRET, ion mobility-mass spectrometry, fluorescence lifetime imaging microscopy

## Abstract

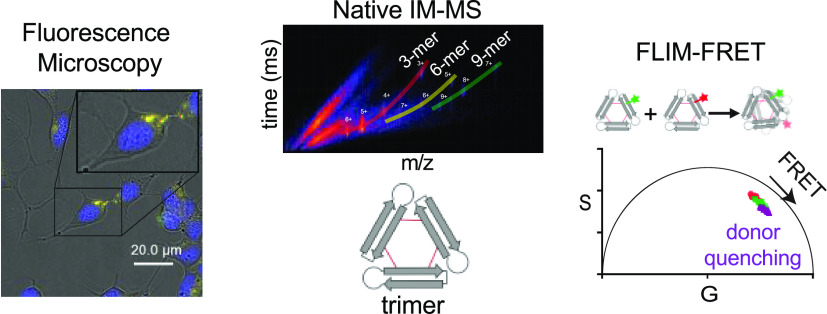

Aβ oligomers play a central role in the neurodegeneration observed with Alzheimer’s disease. Our laboratory has developed covalently stabilized trimers derived from residues 17–36 of Aβ as model systems for studying Aβ oligomers. In the current study, we apply the emerging techniques of fluorescence lifetime imaging microscopy (FLIM) and native mass spectrometry (native MS) to better understand the assembly and interactions of the oligomer model system 2AT-L in aqueous solutions and with cells. 2AT-L and fluorescently labeled 2AT-L analogues assemble in the membrane-like environment of SDS-PAGE, showing diffuse bands of oligomers in equilibrium. Native ion mobility-mass spectrometry (native IM-MS) of 2AT-L allows for the identification of discrete oligomers in solution and shows similar patterns of oligomer formation between 2AT-L and fluorescently labeled analogues. Fluorescence microscopy with SH-SY5Y cells reveals that fluorescently labeled 2AT-L analogues colocalize within lysosomes. FLIM studies with phasor analysis further elucidate the assembly of 2AT-L within cells and establish the occurrence of FRET, indicating the presence of oligomers within cells. Collectively, these multiple complementary techniques help better understand the complex behavior of the 2AT-L model system.

## Introduction

Elucidating the biological properties of Aβ oligomers and understanding their solution-phase behavior are essential to better understanding Alzheimer’s disease.^1^ Accumulation of the β-amyloid peptide (Aβ) in the brain is a hallmark of Alzheimer’s disease and is a key contributor to neurodegeneration.^2^ Aβ aggregates to form toxic oligomers and the fibrils that make up the characteristic plaques observed in the brains from those with Alzheimer’s disease.^3^ These oligomers are thought to be the main synaptotoxic species but have proven difficult to study as they are inherently heterogeneous and metastable with a high propensity to form fibrils.^4−9^ Trimers of Aβ_42_ are among the most toxic oligomers and are implicated in neuronal cell death.^7,10−13^

Model systems of stabilized Aβ-derived oligomers have emerged as tools to better understand endogenous oligomers and provide further insight into the molecular basis of Alzheimer’s disease.^13−15^ Our laboratory has developed a series of covalently stabilized trimers derived from residues 17–36 of Aβ as chemical models of toxic amyloid oligomers associated with neurodegeneration in Alzheimer’s disease.^16−21^ One of the trimers, termed 4AT-L, is composed of three β-hairpins formed by an Aβ_17–36_ peptide, with molecular templates designed to induce β-hairpin folding, block uncontrolled aggregation, and allow disulfide crosslinking of three β-hairpins to form a covalent trimer.^21^ Trimer 4AT-L is toxic toward the neuronal cell line SH-SY5Y, assembles to form ball-shaped dodecamers composed of four trimers in the crystal state, and forms dodecamers in SDS-PAGE.

Fluorescence lifetime imaging microscopy (FLIM) and native mass spectrometry (native MS) have emerged as important new tools to probe the assembly and interactions of peptides and proteins.^22−26^ Native MS provides information about the stoichiometry of oligomeric assemblies present in solution and can thus complement the structural information elucidated from X-ray crystallography and SDS-PAGE to build a broader understanding of the solution-phase assembly of Aβ-derived oligomers.^27,28^ Fluorescence microscopy to visualize Aβ-derived oligomers in the presence of cells and the application of FLIM to determine the occurrence of Förster resonance energy transfer (FRET) can complement the previously observed cellular cytotoxicity.^29−35^ In the current study, we set out to apply these and other techniques to better understand the assembly and interactions of a covalently stabilized trimer derived from residues 17–36 of Aβ in aqueous solutions and with cells.

## Results and Discussion

### Design and Synthesis of 2AT-L

**Figure 1:**
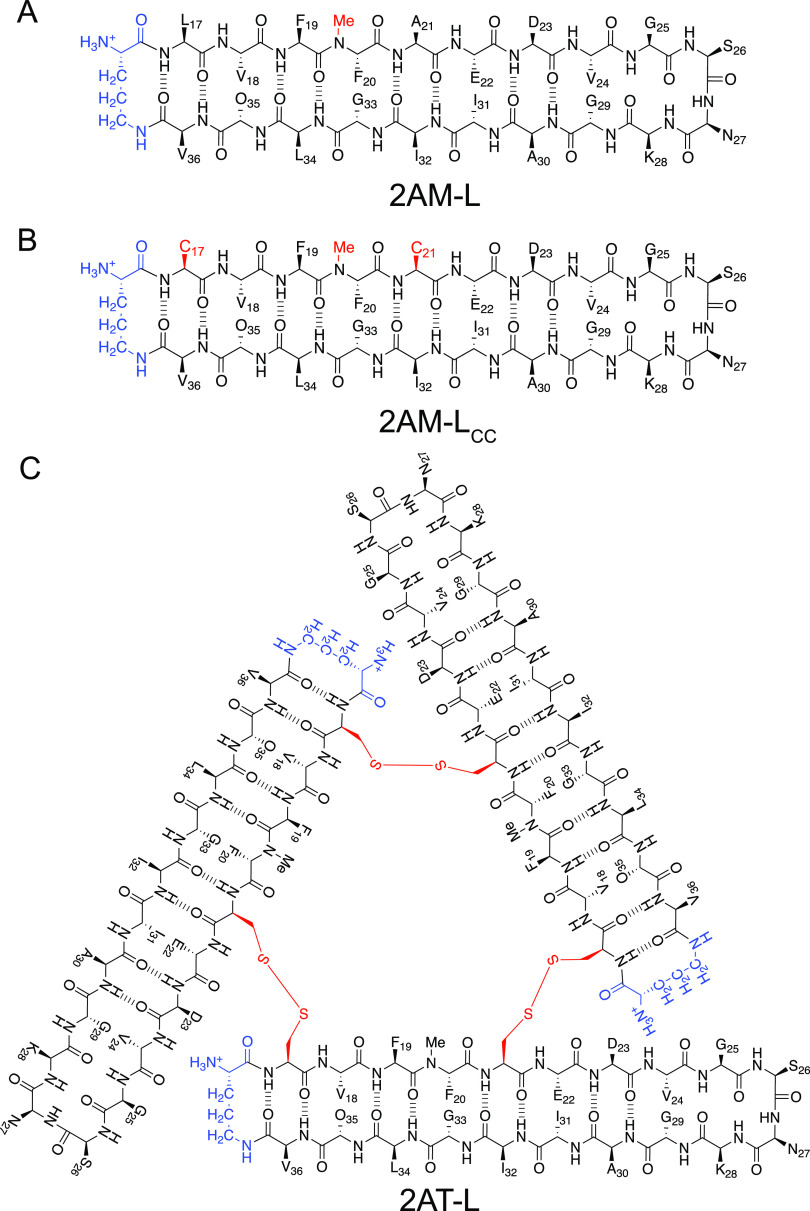
Chemical structures of β-hairpin peptides and a trimer derived from Aβ_17–36_. (A) Macrocyclic β-hairpin 2AM-L incorporating an *N*-methyl residue to prevent uncontrolled aggregation and a ^δ^Orn turn (blue) mimic to constrain the N- and C-termini. (B) β-Hairpin 2AM-L_CC_ incorporates cysteine mutations at residues 17 and 21. (C) Three 2AM-L_CC_ β-hairpins form covalently stabilized trimer 2AT-L through disulfide bonds.

In trimer 4AT-L, the native phenylalanine at position 20 is replaced with cyclohexylalanine. For the current study, we prepared homologue 2AT-L, which behaves similarly but has the native phenylalanine at position 20. Trimer 2AT-L is composed of three crosslinked Aβ_17–36_ β-hairpins. In each β-hairpin, a δ-linked ornithine (^δ^Orn) turn unit between residues 17 and 36 helps enforce a folded β-hairpin conformation, and an *N*-methyl group on phenylalanine 20 helps block uncontrolled aggregation (Figure 1A). In 2AT-L, residues 17 and 21 are replaced by cysteines, which provide covalent crosslinks that hold the trimer together by connecting the monomer subunits at the vertices (Figure 1B,C). The crosslinked trimers are homogeneous, stable, and mimic some of the biological properties of Aβ oligomers.^19,21^

We synthesized trimer 2AT-L by the same procedures that we previously used to prepare trimer 4AT-L. We first prepared macrocyclic peptide 2AM-L_CC_ by solid-phase peptide synthesis of the corresponding linear peptide on 2-chlorotrityl resin, followed by cleavage of the protected peptide from the resin, solution-phase macrocyclization, deprotection, and purification by RP-HPLC. 2AM-L_CC_ was oxidized at 6 mM in 20% aqueous DMSO with 60 mM triethylamine for 48 h. 2AT-L was isolated from the oxidation reaction by RP-HPLC. Pure fractions were lyophilized, affording >95% pure 2AT-L as the trifluoroacetate (TFA) salt.

### Preparation of Fluorescently Labeled 2AT-L

Substoichiometric labeling of 2AT-L with fluorophore NHS esters permits the isolation of singly labeled 2AT-L.^36^ We developed a labeling procedure in which 2AT-L is treated with 0.05 molar equivalents of the commercially available sulfo-cyanine3 (sCy3) or sulfo-cyanine5 (sCy5) NHS esters, and the singly labeled trimer is isolated by RP-HPLC, and the unlabeled trimer is recycled. We found that heating the HPLC column greatly facilitates the separation of the labeled trimer from the unlabeled trimer and also permits the removal of the small amounts of doubly labeled trimer that form. We selected the bis-sulfonated analogues of the commonly used FRET partners Cy3 and Cy5 as fluorophores because they provide enhanced aqueous solubility and reduced aggregation. Labeling 3 mg of trimer by this procedure typically permits isolation of ca. 150 μg of singly labeled trimer 2AT-L-sCy3 or 2AT-L-sCy5 as the TFA salt, which was sufficient for many of the experiments described below.

### Oligomerization of 2AT-L and Fluorescently Labeled 2AT-L Analogues by SDS-PAGE

**Figure 2:**
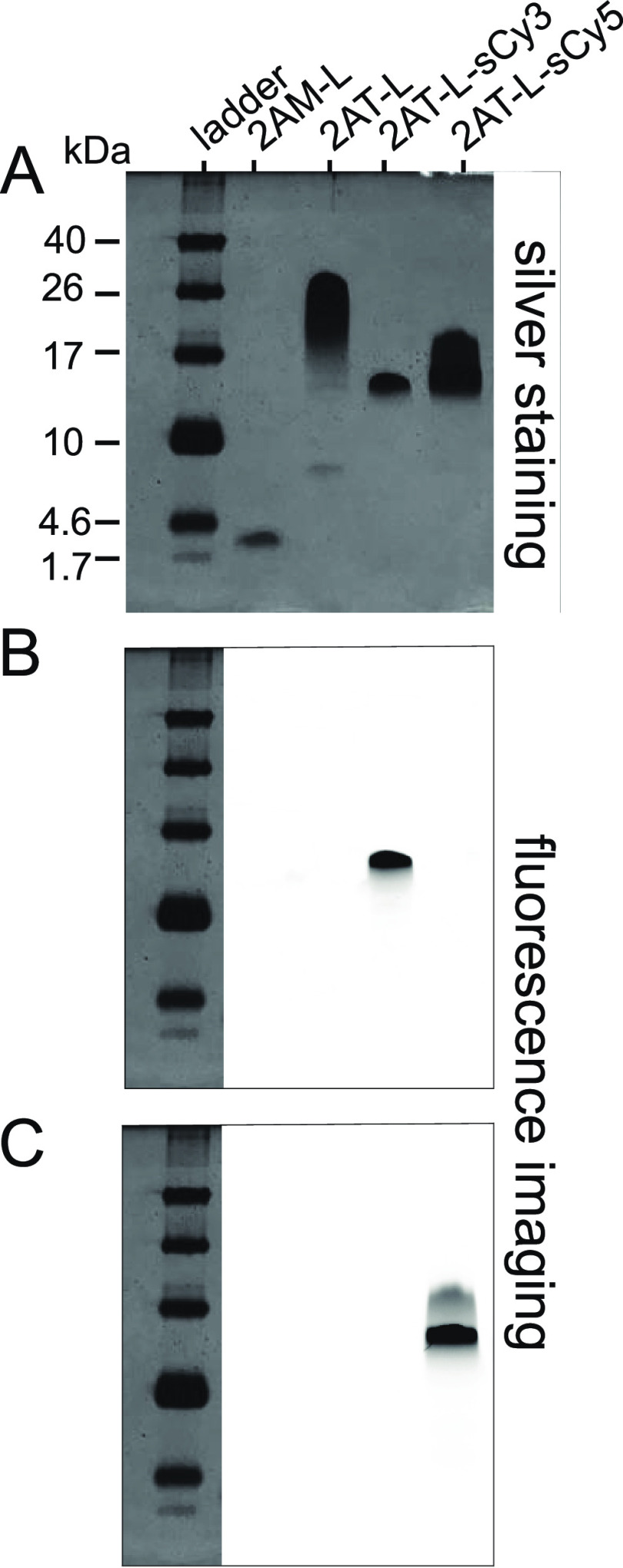
SDS-PAGE of peptides 2AM-L, 2AT-L, 2AT-L-sCy3, and 2AT-L-sCy5. (A) Silver-stained image. (B) Fluorescence image in the Cy3 channel. (C) Fluorescence image in the Cy5 channel. SDS-PAGE was performed in Tris buffer at pH 6.8 with 2% (w/v) SDS on a 16% polyacrylamide gel with 50 μM solutions of peptide in each lane. Fluorescence imaging was performed before silver staining.

We used SDS-PAGE to initially assess the effect of the sCy3 and sCy5 fluorophores on the assembly of 2AT-L into higher-order oligomers. In SDS-PAGE, trimer 2AT-L assembles to form higher-order oligomers. When 2AT-L (6.6 kDa) is run in SDS-PAGE and visualized by silver staining, it forms a downward-streaking band from ca. 32 kDa to ca. 13 kDa, suggesting assembly into oligomers ca. 2–5 trimers in size (Figure 2A). In contrast, the monomer 2AM-L (2.2 kDa) migrates at ca. 3 kDa, indicating the absence of assembly into oligomers. 2AT-L-sCy3 (7.2 kDa) migrates as a compact band at ca. 14 kDa, suggesting hexamer formation (2 trimers), while 2AT-L-sCy5 (7.2 kDa) migrates as a more diffuse band, from ca. 21 kDa to ca. 13 kDa, suggesting the formation of hexamers or perhaps hexamers and nonamers (2–3 trimers).

Fluorescence imaging provides additional insights into the bands formed by the trimers (Figure 2B,C). Notably, the diffuse band formed by 2AT-L-sCy5 shows an intense component at ca. 14 kDa and a weaker component at ca. 21 kDa. The difference in shape between the bands formed by 2AT-L-sCy5 and 2AT-L-sCy3 may reflect the greater hydrophobicity and flat hydrophobic surface area provided by the larger sCy5 fluorophore. Both the sCy3 and sCy5 labels appear to impede the assembly, with hexamers as the main species observed, while the unlabeled 2AT-L forms predominantly larger oligomers.^37^

### Oligomerization of 2AT-L and Fluorescently Labeled 2AT-L Analogues by Native IM-MS

**Figure 3:**
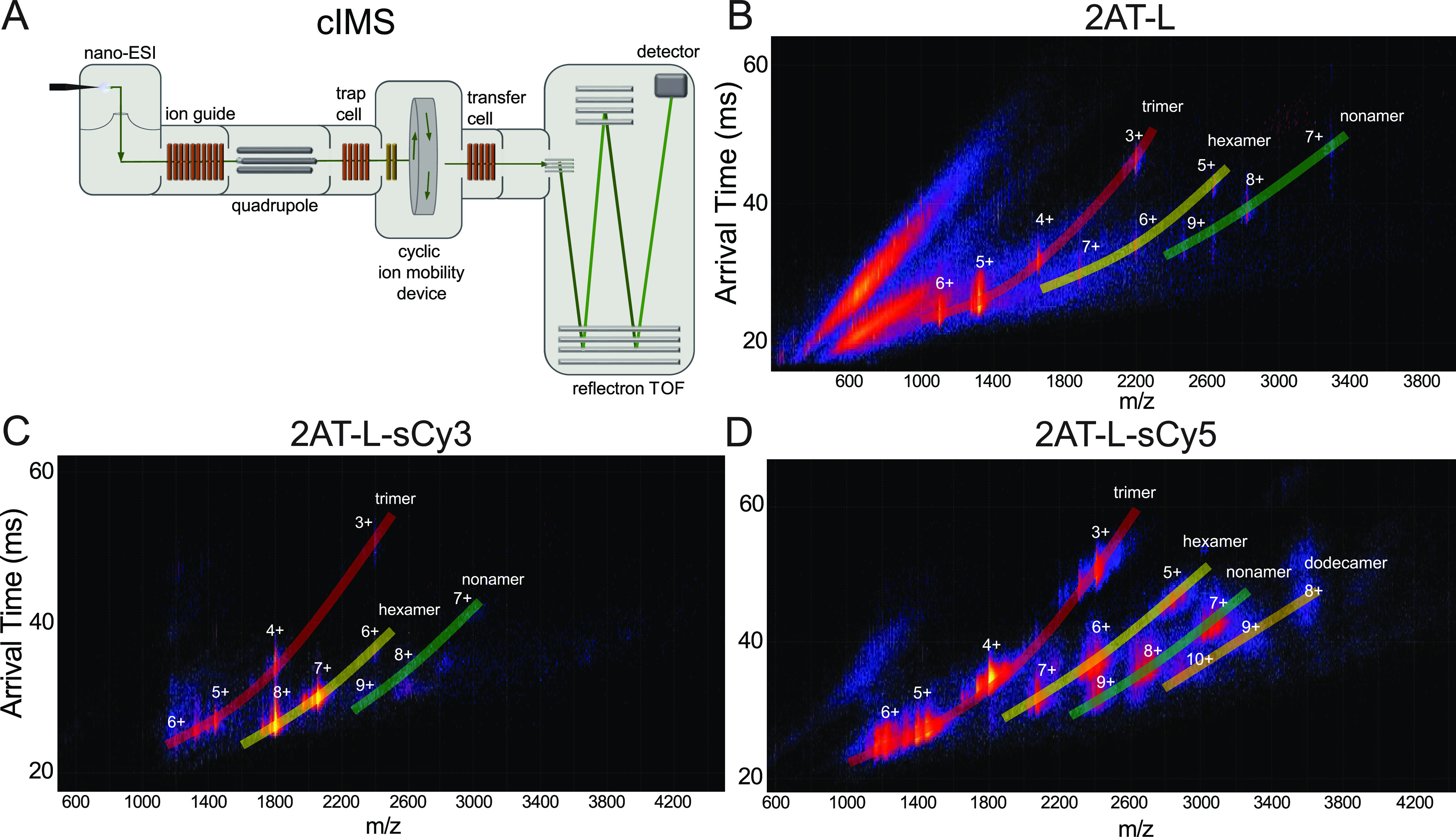
Native IM-MS of 2AT-L and labeled analogues. (A) Diagram of the cIMS instrument used to run native IM-MS experiments. Molecules are ionized through nano-electrospray ionization (nano-ESI) and then travel through an ion guide and quadrupole. Ions reach a trap cell before entering the cyclic ion mobility device in which species are separated by size, shape, and charge. Ions exit the mobility device and proceed through a reflectron time-of-flight (TOF) mass analyzer to separate ions by mass-to-charge ratio. The time for an ion to travel through the mobility cell to detection is recorded as the arrival time. (B–D) Mobiligrams of 2AT-L, 2AT-L-sCy3, and 2AT-L-sCy5. Native IM-MS was performed on a SELECT SERIES Cyclic IMS Q-cIMS-TOF (cIMS) instrument (Waters Corporation). Numeric labels indicate the charge state of the corresponding oligomeric species. Samples of 2AT-L and fluorescently labeled 2AT-L analogues were prepared at a concentration of 40 μM in 400 mM ammonium acetate.

We used native MS coupled with ion mobility spectrometry (IM) to investigate the oligomeric assembly of the trimers to complement the SDS-PAGE studies and avoid SDS-induced oligomer formation.^38^ In native IM-MS, oligomers and noncovalent assemblies are ionized and separated without dissociation.^39^ IM separates ionized oligomers of different sizes, shapes, and charges in the gas phase as they travel through the ion mobility device at different rates, and their arrival times are measured.^40^ The ions then are analyzed by a mass analyzer to determine their mass-to-charge ratio, *m*/*z* (Figure 3A). Trimer 2AT-L and fluorescently labeled 2AT-L analogues show a charge state distribution in the mass spectrum, leading to many overlapping *m*/*z* peaks that arise from multiple assemblies of trimers. The separation by arrival times that occurs in the ion mobility device permits the separation of species with identical mass-to-charge ratios into their component oligomers.

Native IM-MS reveals that 2AT-L and fluorescently labeled 2AT-L analogues also assemble to form oligomers in aqueous solution. Each IM-MS experiment generates a mobiligram, in which the arrival time is plotted against mass-to-charge ratio, with the relative intensity displayed as a heat map. The IM-MS mobiligram for trimer 2AT-L shows that 2AT-L forms hexamers and nonamers, in addition to the trimer (Figure 3B). IM-MS shows that 2AT-L-sCy3 and 2AT-L-sCy5 also undergo assembly. The mobiligram for 2AT-L-sCy3 shows the formation of hexamers, as well as a low abundance of nonamers and dodecamers, in addition to the trimer (Figure 3C). The mobiligram for 2AT-L-sCy5 also shows the formation of hexamers, nonamers, and dodecamers, in addition to the trimer (Figure 3D).^41^

Together, the SDS-PAGE and native IM-MS experiments reveal that oligomers of 2AT-L and the fluorescently labeled 2AT-L analogues can form in either the presence or absence of SDS. Although the fluorophore labels appear to perturb the formation of higher-order oligomers in SDS-PAGE, a similar perturbation is not seen in native IM-MS.

### Co-Oligomerization of 2AT-L-sCy3 and 2AT-L-sCy5 by FRET

We performed steady-state FRET experiments with mixtures of 2AT-L-sCy3 (donor) and 2AT-L-sCy5 (acceptor) to further assess the assembly of 2AT-L into higher-order oligomers in solution. The occurrence of FRET can be determined by selectively exciting at a wavelength absorbed by the donor and observing fluorescence at a wavelength emitted by the acceptor. In practice, it is often difficult to selectively excite only the donor. Thus, we used an excitation wavelength of 490 nm—which is well below the 548 nm λ_max_ of sCy3—to minimize direct excitation of sCy5.^42^ We monitored fluorescence emission at 662 nm—the emission maximum of sCy5—and varied the ratio of 2AT-L-sCy3 and 2AT-L-sCy5 from 100:0 to 0:100 while maintaining a total concentration of 5 μM.

**Figure 4:**
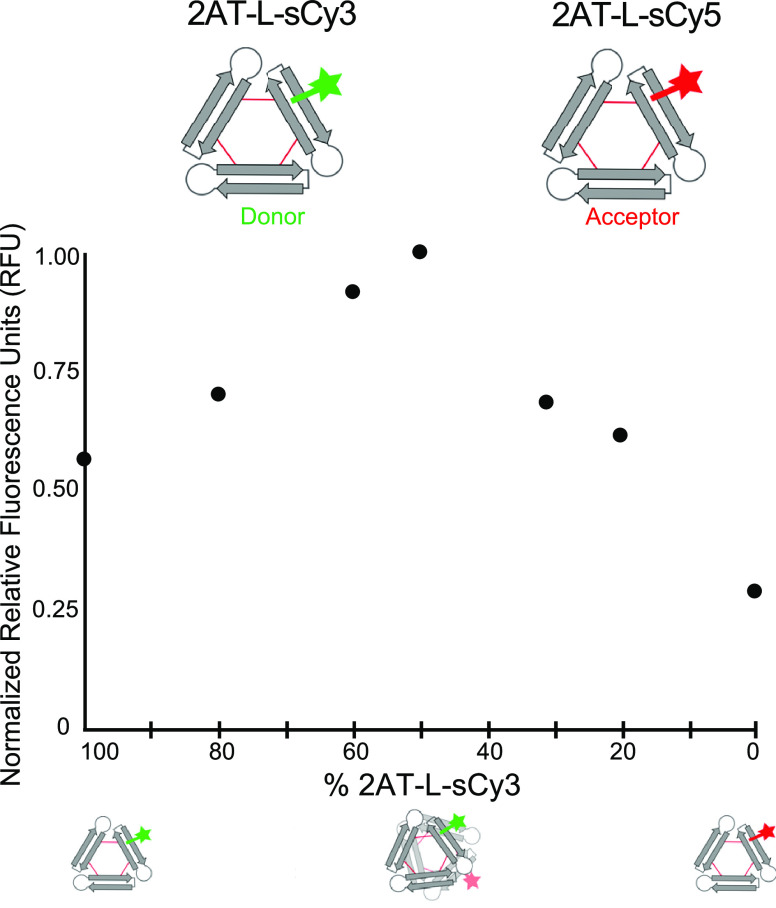
Fluorescence at the emission maximum of 2AT-L-sCy5 as a function of the percentage of 2AT-L-sCy3. Fluorescence spectra of mixtures of varying ratios of 2AT-L-sCy3 and 2AT-L-sCy5 were acquired with excitation at 490 nm and observation at 662 nm. All spectra were collected on aqueous solutions of 2AT-L-sCy3 and 2AT-L-sCy5 with a total concentration of 5 μM.

When 2AT-L-sCy3 alone (100:0) is irradiated at 490 nm, emission (0.57 rfu) occurs at 662 nm (Figure 4). This emission results from the sCy3 fluorophore, which has an emission maximum of 556 nm, but which extends weakly to 662 nm and beyond. When a 50:50 mixture of 2AT-L-sCy3 and 2AT-L-sCy5 is irradiated, the emission at 662 nm increases (1.00 rfu). The enhanced fluorescence reflects the occurrence of FRET and thus demonstrates the co-oligomerization of 2AT-L-sCy3 and 2AT-L-sCy5. Only modest emission (0.29 rfu) occurs from excitation of 2AT-L-sCy5 alone (0:100) at 490 nm, providing further evidence that the enhanced fluorescence in the 50:50 mixture results from FRET. Additional mixtures of 2AT-L-sCy3 and 2AT-L-sCy5 (80:20, 60:40, 40:60, 20:80) show intermediate levels of emission, providing further evidence for co-oligomerization and FRET.

### Fluorescence Microscopy of 2AT-L-sCy3 and 2AT-L-sCy5

To gain insights into the biological roles of the oligomers observed by SDS-PAGE, native IM-MS, and steady-state FRET experiments, we performed fluorescence microscopy with SH-SY5Y cells. Trimer 4AT-L is toxic toward SH-SY5Y cells, and trimer 2AT-L exhibits similar toxicity.^21^ To further explore the interaction of Aβ-derived trimers with mammalian cells, we used fluorescence microscopy. SH-SY5Y cells were incubated with fluorescent analogues of 2AT-L at a total concentration of 5 μM. Cells were then counterstained with Hoechst 33342 nuclear stain (blue), washed with phenol red-free DMEM:F12 media, and imaged. Fluorescence micrographs were collected with emissions observed in the respective Cyanine3 and Cyanine5 channels.

**Figure 5:**
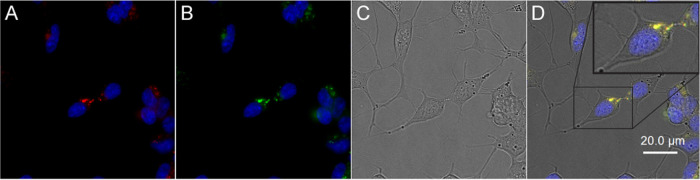
Micrographs illustrating intracellular co-localization of fluorescently labeled 2AT-L analogues in SH-SY5Y cells. (A) 2AT-L-sCy5 (red) and Hoechst 33342 nuclear stain (blue). (B) 2AT-L-sCy3 (green) and Hoechst 33342 nuclear stain (blue). (C) Brightfield image. (D) Merged fluorescent images with brightfield images. Cells were incubated with 5 μM 2AT-L-sCy3 and 5 μM 2AT-L-sCy5 for 6 h at 37°C, counterstained with Hoechst 33342 nuclear stain, and imaged.

Fluorescence microscopy reveals that both 2AT-L-sCy3 and 2AT-L-sCy5 are internalized by cells. Incubation of SH-SY5Y cells with 2AT-L-sCy3 resulted in defined intracellular puncta and some small fluorescent features bound to the cell membrane (Figure S2). Incubation of SH-SY5Y cells with 2AT-L-sCy5 resulted in similar features (Figure S3). Incubation of SH-SY5Y cells with both 2AT-L-sCy3 and 2AT-L-sCy5 also resulted in similar features (Figure 5). Merged images, showing both the green (2AT-L-sCy3) and red (2AT-L-sCy5) channels, show a yellow hue, indicating co-localization of the fluorescently labeled trimers. Punctate features were observed as early as 5 h after treatment and at concentrations of fluorescently labeled 2AT-L as low as 8 nM (Figure S4).

The punctate intracellular features observed for the labeled 2AT-L are similar to those reported for fluorescently labeled Aβ_42_, where uptake occurred primarily through endocytosis.^43−50^ These studies have shown that Aβ localizes in lysosomes. To assess whether 2AT-L also localizes into lysosomes, we performed further experiments using the lysosomal marker Lysotracker Green. Treatment of SH-SY5Y cells with Lysotracker Green and either 2AT-L-sCy3 or 2AT-L-sCy5 showed co-localization, indicating sequestration in the lysosomes (Figures S5 and 6). Thus, it appears that the uptake of 2AT-L occurs through endocytosis, in a fashion similar to that of Aβ.

**Figure 6:**
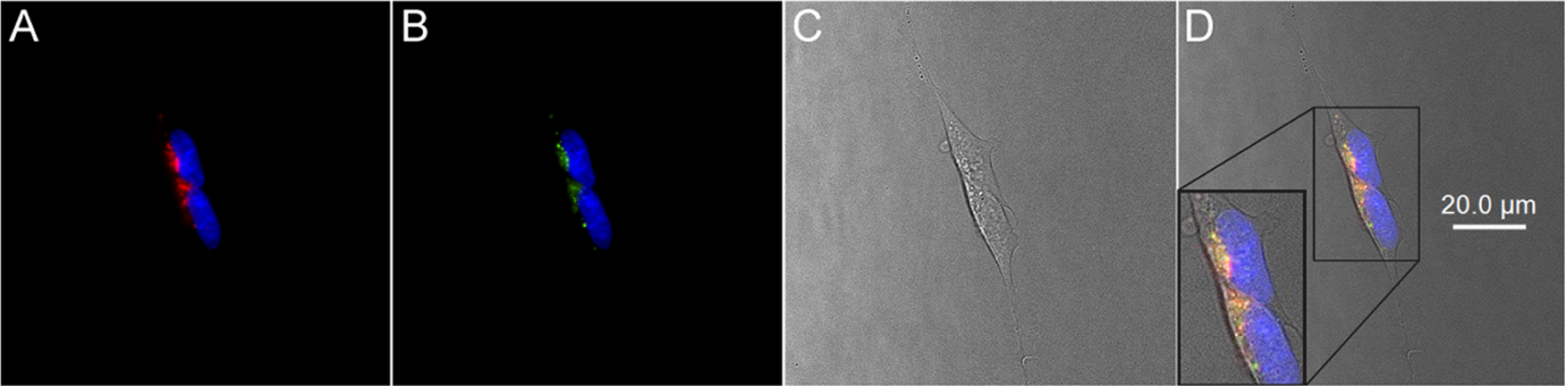
Micrographs illustrating intracellular co-localization of 2AT-L-sCy5 and Lysotracker Green in SH-SY5Y cells. (A) 2AT-L-sCy5 (red) and Hoechst 33342 nuclear stain (blue). (B) Lysotracker Green (green) and Hoechst 33342 nuclear stain (blue). (C) Brightfield image. (D) Merged fluorescent images with brightfield images. Cells were incubated with 5 μM 2AT-L-sCy5 and 100 nM Lysotracker Green for 6 h at 37°C, counterstained with Hoechst 33342 nuclear stain, and imaged.

Previous reports have suggested that oligomerization is a prerequisite for the cellular uptake of Aβ.^51^ The covalently stabilized oligomer model 2AT-L is readily taken up by cells and may thus serve as a model to further study the cellular uptake of Aβ oligomers. To continue to probe the assembly state of 2AT-L-sCy3 and 2AT-L-sCy5 and assess for higher-order oligomer formation, we turned to FRET microscopy.

### FLIM-FRET Studies of 2AT-L-sCy3 and 2AT-L-sCy5

We used fluorescence lifetime imaging microscopy (FLIM) to further investigate the intracellular puncta observed when fluorescently labeled 2AT-L analogues are incubated with cells. FLIM allows the detection of FRET and can thus reveal molecular co-localization at a resolution higher than can be achieved through confocal microscopy. If 2AT-L molecules bearing FRET partners co-assemble or are otherwise in close proximity (<10 nm), FRET can occur and will manifest as a reduction in the fluorescence lifetime of the FRET donor through fluorescence quenching.^52^ Measurement of fluorescence lifetimes through FLIM-FRET offers advantages over intensity-based FRET measurements because the measurement of the lifetime is generally independent of the concentration of the fluorophores and does not require correction for spectral crosstalk.^53^

**Figure 7:**
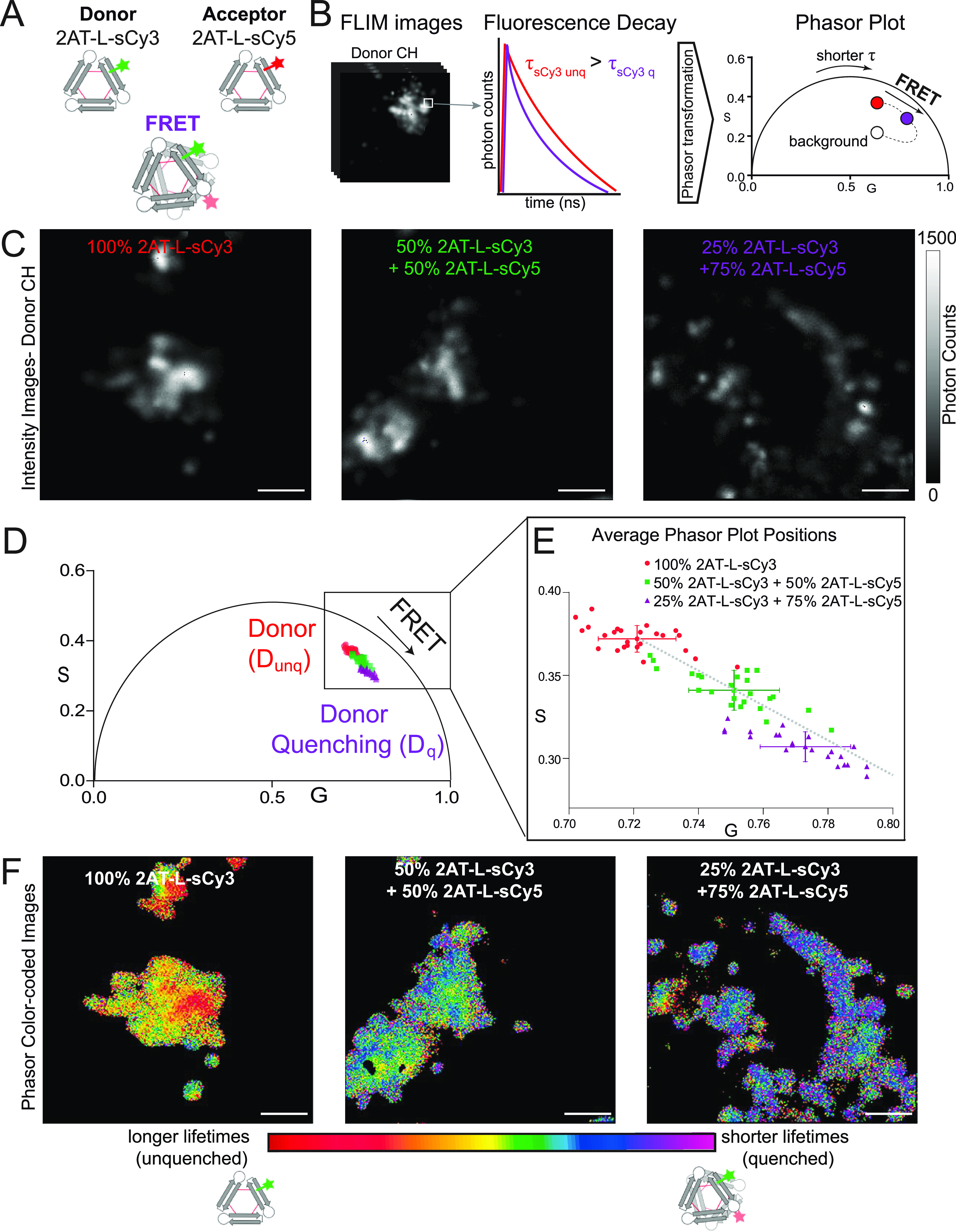
Phasor analysis of FLIM micrographs of SH-SY5Y cells treated with 2AT-L-sCy3 and 2AT-L-sCy5. Oligomeric assemblies of 2AT-L-sCy3 and 2AT-L-sCy5 within cells were analyzed by FLIM-FRET and the phasor approach. (A) Representation of the co-assembly of 2AT-L-sCy3 (donor) and 2AT-L-sCy5 (acceptor) and the occurrence of FRET. (B) Representation of the phasor transformation of FLIM images. Fluorescence lifetime of each pixel of each image is analyzed and plotted on a phasor plot. When FRET occurs, the excited state lifetime of the donor decreases and the phasor position shifts toward shorter lifetimes. The curved FRET trajectory (dotted line) follows the classical definition of FRET efficiency and is influenced by the background fluorescence lifetime. (C) Representative images showing photon intensity of SH-SY5Y cells treated with 2AT-L-sCy3 and 2AT-L-sCy5 in 100:0, 50:50, and 25:75 ratios. Images were acquired in the donor (sCy3) channel. Scale bar = 5 μm. (*D*) Phasor plot illustrating the average phasor position in each image. Images collected for the 100:0 ratio are represented by red points and correspond to unquenched donor (*D*_unq_). Images collected for the 50:50 ratio are represented by green points. Images collected for the 25:75 ratio are represented by violet points and correspond to increased donor quenching (*D*_q_). (E) Details of phasor plot in panel (D). Average phasor plot positions (±SD) from each image. (F) Representative images of SH-SY5Y cells with false color illustrating fluorescence lifetime. Longer fluorescence lifetimes are represented in red and orange, while shorter fluorescence lifetimes reflecting increased FRET are represented progressively in yellow, green, blue, and violet.

To assess FRET inside cells, we treated SH-SY5Y cells with either 2AT-L-sCy3 (donor) alone or with mixtures of 2AT-L-sCy5 (acceptor) and 2AT-L-sCy3 and looked for decreases in the fluorescence lifetime of sCy3 with increasing mole fraction of sCy5. Although 2AT-L-sCy3 oligomers or 2AT-L-sCy5 oligomers can form, only oligomers containing both 2AT-L-sCy3 and 2AT-L-sCy5 are expected to show decreased fluorescence lifetime (Figure 7A).^54^ We used the phasor approach to analyze changes in the fluorescence lifetime of sCy3. In this method, each pixel of a FLIM image is transformed to a point on a phasor plot, allowing for simple visualization of changes in the donor lifetime in a micrograph and thus facilitating the assessment of FRET.^55−58^ Changes in fluorescence lifetime with increases in mole fraction of sCy5 manifest as changes in the positions of points on the phasor plot (Figure 7B), with greater changes corresponding to a greater fraction of molecules undergoing FRET.^55,59,60^

We performed three sets of experiments, in which SH-SY5Y cells were treated with 2AT-L-sCy3 and 2AT-L-sCy5 in 100:0, 50:50, and 25:75 ratios while maintaining a total concentration of 5 μM. For each ratio, we collected 25–26 images of individual cells. We observed the photon intensity in the donor channel decrease as the fraction of 2AT-L-sCy5 increased (Figure 7C). The fluorescence decay from each pixel was determined, and the average from each image was plotted onto a phasor plot, with different colors (red, green, and violet) representing the three sets of ratios studied (Figure 7D,E). The resulting phasor plot shows significant decreases in fluorescence lifetime with an increasing fraction of sCy5 and thus provides compelling evidence for FRET and hence intimate proximity of 2AT-L-sCy3 and 2AT-L-sCy5 in the cells.

The fluorescence lifetimes measured in FLIM-FRET can also be represented as a heat map, with each pixel of an image colored to reflect its relative fluorescence lifetime. We have thus represented the distribution of fluorescence lifetimes on three representative images at the 100:0, 50:50, and 25:75 ratios, with red representing the longest lifetimes and violet representing the shortest lifetimes, and colors ranging from orange to blue representing intermediate lifetimes (Figure 7F). The shift from red toward violet across the series of images reflects the decrease in fluorescence lifetime with an increasing fraction of 2AT-L-sCy5 and further demonstrates that FRET increases with the addition of sCy5. Collectively, these FLIM-FRET experiments provide further evidence that 2AT-L-sCy3 and 2AT-L-sCy5 co-oligomerize in cells.

## Summary and Conclusions

Our covalently stabilized Aβ-derived trimers mimic some of the assembly and biological properties of Aβ oligomers. In the crystal state, 2AT-L and homologue 4AT-L assemble to form ball-shaped dodecamers. In the membrane-like environment of SDS-PAGE, 2AT-L dodecamers are also observed but appear to be in equilibrium with oligomers ca. 2–5 trimers in size. Labeling 2AT-L with sCy3 and sCy5 appears to attenuate assembly in SDS-PAGE, with 2AT-L-sCy3 and 2AT-L-sCy5 migrating mainly as hexamers. Native IM-MS of 2AT-L identifies discrete hexamers and nonamers in solution. Native IM-MS of 2AT-L-sCy3 and 2AT-L-sCy5 shows similar oligomer formation to 2AT-L.^61^

Steady-state FRET experiments also show that 2AT-L-sCy3 and 2AT-L-sCy5 form oligomers in solution. Fluorescence microscopy shows that 2AT-L-sCy3 and 2AT-L-sCy5 form intracellular puncta in SH-SY5Y cells, which co-localize with a lysosomal marker, suggesting an uptake mechanism through endocytotic vesicles that is consistent with previous studies of Aβ.^49^ FLIM studies and phasor analysis establish the occurrence of FRET within cells treated with both 2AT-L-sCy3 and 2AT-L-sCy5, suggesting oligomerization within cellular vesicles.

The aggregation of Aβ to form oligomers is difficult to study in detail due to the complexity of the different species that form and the propensity of Aβ to ultimately form insoluble fibrils. The constraint of an Aβ-derived peptide into a stable trimer, 2AT-L, provides a model system that is easier to study and can be explored by a variety of biophysical and biological techniques. While X-ray crystallography provides a simple answer that assemblies of dodecamers form in the crystal state, the behavior in the solution state is more complex. SDS-PAGE shows multiple oligomeric states in equilibrium but does not allow the identification of discrete oligomers. Native IM-MS, on the other hand, allows the identification of individual oligomers. The addition of fluorescent labels is required to visualize the interactions of 2AT-L in cells, although SDS-PAGE shows that these labels can alter the oligomerization, at least somewhat. FLIM-FRET experiments with phasor analysis allow the observation of oligomerization of the labeled 2AT-L in cells. Collectively, the application of multiple complementary techniques helps better elucidate the complex behavior of the 2AT-L model system. We anticipate that these techniques may also be useful for understanding the yet more complex oligomerization of Aβ.

## Materials and Methods

Peptides 2AM-L, 2AM-L_CC_, and 2AT-L were synthesized by procedures analogous to those described previously.^19^ SDS-PAGE and silver staining were performed as described previously.^21^ Procedures detailing the preparation of 2AT-L-sCy3 and 2AT-L-sCy5, native mass spectrometry studies, steady-state FRET experiments, fluorescence microscopy, and FLIM-FRET can be found in the Supporting Information.

## Supporting Information Available

Procedures detailing the preparation of 2AT-L-sCy3 and 2AT-L-sCy5, native mass spectrometry studies, steady-state FRET experiments, fluorescence microscopy, and FLIM-FRET (PDF)

## References

[ref1] Mroczko B.; Groblewska M.; Litman-Zawadzka A.; Kornhuber J.; Lewczuk P. Amyloid β Oligomers (AβOs) in Alzheimer’s Disease. J. Neural Transm. 2018, 125, 177–191. 10.1007/s00702-017-1820-x. 29196815

[ref2] Pearson H. A.; Peers C. Physiological Roles for Amyloid β Peptides. J. Physiol. 2006, 575, 5–10. 10.1113/jphysiol.2006.111203. 16809372 PMC1819417

[ref3] Nguyen P. H.; Ramamoorthy A.; Sahoo B. R.; Zheng J.; Faller P.; Straub J. E.; Dominguez L.; Shea J. E.; Dokholyan N. V.; de Simone A.; Ma B.; Nussinov R.; Najafi S.; Ngo S. T.; Loquet A.; Chiricotto M.; Ganguly P.; McCarty J.; Li M. S.; Hall C.; Wang Y.; Miller Y.; Melchionna S.; Habenstein B.; Timr S.; Chen J.; Hnath B.; Strodel B.; Kayed R.; Lesné S.; Wei G.; Sterpone F.; Doig A. J.; Derreumaux P. Amyloid Oligomers: A Joint Experimental/Computational Perspective on Alzheimer’s Disease, Parkinson’s Disease, Type II Diabetes, and Amyotrophic Lateral Sclerosis. Chem. Rev. 2021, 121, 2545–2647. 10.1021/acs.chemrev.0c01122. 33543942 PMC8836097

[ref4] Ono K.; Condron M. M.; Teplow D. B. Structure-Neurotoxicity Relationships of Amyloid β-Protein Oligomers. Proc. Natl. Acad. Sci. U.S.A. 2009, 106, 14745–14750. 10.1073/pnas.0905127106. 19706468 PMC2736424

[ref5] Gray A. L. H.; Sawaya M. R.; Acharyya D.; Lou J.; Edington E.; Best M. D.; Prosser R. A.; Eisenberg D. S.; Do T. D. Atomic View of an Amyloid Dodecamer Exhibiting Selective Cellular Toxic Vulnerability in Acute Brain Slices. Protein Sci. 2021, 106, 1–12. 10.1002/pro.4268. PMC8862425 34954854

[ref6] Sakono M.; Zako T. Amyloid Oligomers: Formation and Toxicity of Aβ Oligomers. FEBS J. 2010, 277, 1348–1358. 10.1111/j.1742-4658.2010.07568.x. 20148964

[ref7] Walsh D. M.; Selkoe D. J. Aβ Oligomers - A Decade of Discovery. J. Neurochem. 2007, 101, 1172–1184. 10.1111/j.1471-4159.2006.04426.x. 17286590

[ref8] LaFerla F. M.; Green K. N.; Oddo S. Intracellular Amyloid-β in Alzheimer’s Disease. Nat. Rev. Neurosci. 2007, 8, 499–509. 10.1038/nrn2168. 17551515

[ref9] Cline E. N.; Bicca M. A.; Viola K. L.; Klein W. L. The Amyloid-Oligomer Hypothesis: Beginning of the Third Decade. J. Alzheimers Dis. 2018, 64, 567–610. 10.3233/JAD-179941. PMC6004937 29843241

[ref10] Jana M. K.; Cappai R.; Pham C. L. L.; Ciccotosto G. D. Membrane-Bound Tetramer and Trimer Aβ Oligomeric Species Correlate with Toxicity towards Cultured Neurons. J. Neurochem. 2016, 136, 594–608. 10.1111/jnc.13443. 26608930

[ref11] Marina G. B.; Kirkitadze D.; Lomakin A.; Vollers S. S.; Benedek G. B.; Teplow D. B. Amyloid β-Protein (Aβ) Assembly: Aβ40 and Aβ42 Oligomerize through Distinct Pathways. Proc. Natl. Acad. Sci. U.S.A. 2003, 100, 330–335. 10.1073/pnas.222681699. 12506200 PMC140968

[ref12] Cleary J. P.; Walsh D. M.; Hofmeister J. J.; Shankar G. M.; Kuskowski M. A.; Selkoe D. J.; Ashe K. H. Natural Oligomers of the Amyloid-β Protein Specifically Disrupt Cognitive Function. Nat. Neurosci. 2005, 8, 79–84. 10.1038/nn1372. 15608634

[ref13] Benilova I.; Karran E.; De Strooper B. The Toxic Aβ Oligomer and Alzheimer’s Disease: An Emperor in Need of Clothes. Nat. Neurosci. 2012, 15, 349–357. 10.1038/nn.3028. 22286176

[ref14] Hawk L. M. L.; Pittman J. M.; Moore P. C.; Srivastava A. K.; Zerweck J.; Williams J. T. B.; Hawk A. J.; Sachleben J. R.; Meredith S. C. β-Amyloid Model Core Peptides: Effects of Hydrophobes and Disulfides. Protein Sci. 2020, 29, 527–541. 10.1002/pro.3778. 31710741 PMC6954707

[ref15] Straub J. E.; Thirumalai D. Principles Governing Oligomer Formation in Amyloidogenic Peptides. Curr. Opin. Struct. Biol. 2010, 20, 187–195. 10.1016/j.sbi.2009.12.017. 20106655 PMC2854190

[ref16] Kreutzer A. G.; Nowick J. S. Elucidating the Structures of Amyloid Oligomers with Macrocyclic β-Hairpin Peptides: Insights into Alzheimer’s Disease and Other Amyloid Diseases. Acc. Chem. Res. 2018, 51, 706–718. 10.1021/acs.accounts.7b00554. 29508987 PMC5911177

[ref17] Kreutzer A. G.; Spencer R. K.; McKnelly K. J.; Yoo S.; Hamza I. L.; Salveson P. J.; Nowick J. S. A Hexamer of a Peptide Derived from Aβ16-36. Biochemistry 2017, 56, 6061–6071. 10.1021/acs.biochem.7b00831. 29028351 PMC5689071

[ref18] Wang Y.; Kreutzer A. G.; Truex N. L.; Nowick J. S. A Tetramer Derived from Islet Amyloid Polypeptide. J. Org. Chem. 2017, 82, 7905–7912. 10.1021/acs.joc.7b01116. 28661686 PMC7532939

[ref19] Kreutzer A. G.; Yoo S.; Spencer R. K.; Nowick J. S. Stabilization, Assembly, and Toxicity of Trimers Derived from Aβ. J. Am. Chem. Soc. 2017, 139, 966–975. 10.1021/jacs.6b11748. 28001392 PMC5256483

[ref20] Kreutzer A. G.; Samdin T. D.; Guaglianone G.; Spencer R. K.; Nowick J. S. X-Ray Crystallography Reveals Parallel and Antiparallel β-Sheet Dimers of a β-Hairpin Derived from Aβ16-36that Assemble to Form Different Tetramers. ACS Chem. Neurosci. 2020, 11, 2340–2347. 10.1021/acschemneuro.0c00290. 32584538 PMC7811405

[ref21] Haerianardakani S.; Kreutzer A. G.; Salveson P. J.; Samdin T. D.; Guaglianone G. E.; Nowick J. S. Phenylalanine Mutation to Cyclohexylalanine Facilitates Triangular Trimer Formation by β-Hairpins Derived from Aβ. J. Am. Chem. Soc. 2020, 142, 20708–20716. 10.1021/jacs.0c09281. 33237748 PMC7821965

[ref22] Hu J.; Zheng Q. Applications of Mass Spectrometry in the Onset of Amyloid Fibril Formation: Focus on the Analysis of Early-Stage Oligomers. Front. Chem. 2020, 8, 324 10.3389/fchem.2020.00324. 32432078 PMC7215083

[ref23] Ben-Nissan G.; Sharon M. The Application of Ion-Mobility Mass Spectrometry for Structure/Function Investigation of Protein Complexes. Curr. Opin. Chem. Biol. 2018, 42, 25–33. 10.1016/j.cbpa.2017.10.026. 29128665 PMC5796646

[ref24] Matuszyk M. M.; Garwood C. J.; Ferraiuolo L.; Simpson J. E.; Staniforth R. A.; Wharton S. B. Biological and Methodological Complexities of Beta-Amyloid Peptide: Implications for Alzheimer’s Disease Research. J. Neurochem. 2022, 160, 434–453. 10.1111/jnc.15538. 34767256

[ref25] Ueda H. H.; Nagasawa Y.; Murakoshi H. Imaging Intracellular Protein Interactions/Activity in Neurons Using 2-Photon Fluorescence Lifetime Imaging Microscopy. Neurosci. Res. 2021, 179, 31–38. 10.1016/j.neures.2021.10.004. 34666101

[ref26] Aliyan A.; Cook N. P.; Martí A. A. Interrogating Amyloid Aggregates Using Fluorescent Probes. Chem. Rev. 2019, 119, 11819–11856. 10.1021/acs.chemrev.9b00404. 31675223

[ref27] Leney A. C.; Heck A. J. R. Native Mass Spectrometry: What Is in the Name?. J. Am. Soc. Mass Spectrom. 2017, 28, 5–13. 10.1007/s13361-016-1545-3. PMC5174146 27909974

[ref28] Heck A. J. R. Native Mass Spectrometry: A Bridge between Interactomics and Structural Biology. Nat. Methods 2008, 5, 927–933. 10.1038/nmeth.1265. 18974734

[ref29] Bacskai B. J.; Skoch J.; Hickey G. A.; Allen R.; Hyman B. T. Fluorescence Resonance Energy Transfer Determinations Using Multiphoton Fluorescence Lifetime Imaging Microscopy to Characterize Amyloid-Beta Plaques. J. Biomed. Opt. 2003, 8, 368–375. 10.1117/1.1584442. 12880341

[ref30] Clegg R. M. Chapter 1 Förster Resonance Energy Transfer—FRET What Is It, Why Do It, and How It’s Done. Lab. Tech. Biochem. Mol. Biol. 2009, 33, 1–57. 10.1016/S0075-7535(08)00001-6.

[ref31] Jones G. A.; Bradshaw D. S. Resonance Energy Transfer: From Fundamental Theory to Recent Applications. Front. Phys. 2019, 7, 100 10.3389/fphy.2019.00100.

[ref32] Ma L.; Yang F.; Zheng J. Application of Fluorescence Resonance Energy Transfer in Protein Studies. J. Mol. Struct. 2014, 1077, 87–100. 10.1016/j.molstruc.2013.12.071. 25368432 PMC4215735

[ref33] Lou J.; Scipioni L.; Wright B. K.; Bartolec T. K.; Zhang J.; Masamsetti V. P.; Gaus K.; Gratton E.; Cesare A. J.; Hinde E. Phasor Histone FLIM-FRET Microscopy Quantifies Spatiotemporal Rearrangement of Chromatin Architecture during the DNA Damage Response. Proc. Natl. Acad. Sci. 2019, 116, 7323–7332. 10.1073/PNAS.1814965116. 30918123 PMC6462080

[ref34] Ishikawa-ankerhold H. C.; Ankerhold R.; Drummen G. P. C.; Biology C.; Zeiss C.; Gmbh M.; Program B.; Stress C.; Program A. Advanced Fluorescence Microscopy Techniques—FRAP, FLIP, FLAP, FRET and FLIM. Molecules 2012, 17, 4047–4132. 10.3390/molecules17044047. 22469598 PMC6268795

[ref35] Chen Y.; Mills J. D.; Periasamy A. Protein Localization in Living Cells and Tissues Using FRET and FLIM. Differentiation 2003, 71, 528–541. 10.1111/j.1432-0436.2003.07109007.x. 14686950

[ref36] We chose to singly-label trimer 2AT-L to minimize perturbation of its assembly and interactions with cells.

[ref37] A mixture of 2AT-L-SCy3 and 2AT-L-SCy5 was studied by SDS-PAGE and can be found in Figure S1.

[ref38] Pujol-Pina R.; Vilaprinyó-Pascual S.; Mazzucato R.; Arcella A.; Vilaseca M.; Orozco M.; Carulla N. SDS-PAGE Analysis of Aβ Oligomers Is Disserving Research into Alzheimer’s Disease: Appealing for ESI-IM-MS. Sci. Rep. 2015, 5, 14809 10.1038/srep14809. 26450154 PMC4598734

[ref39] Cawood E. E.; Karamanos T. K.; Wilson A. J.; Radford S. E. Visualizing and Trapping Transient Oligomers in Amyloid Assembly Pathways. Biophys. Chem. 2021, 268, 106505 10.1016/j.bpc.2020.106505. 33220582 PMC8188297

[ref40] Snyder D. T.; Jones B. J.; Lin Y. F.; Cooper-Shepherd D. A.; Hewitt D.; Wildgoose J.; Brown J. M.; Langridge J. I.; Wysocki V. H. Surface-Induced Dissociation of Protein Complexes on a Cyclic Ion Mobility Spectrometer. Analyst 2021, 146, 6861–6873. 10.1039/d1an01407b. 34632987 PMC8574189

[ref41] We attempted to study the co-oligomerization of 2AT-L-sCy3 and 2AT-L-sCy5 by IM-MS but did not observe sufficient ionization.

[ref42] Miyawaki A.; Tsien R. Y. Monitoring Protein Conformations and Monitoring Protein Conformations and Interactions by Fluorescence Resonance Energy Transfer between Mutants of Green Fluorescent Protein. Methods Enzymol. 2000, 327, 472–500. 10.1016/s0076-6879(00)27297-2. 11045004

[ref43] Dutta S.; Finn T. S.; Kuhn A. J.; Abrams B.; Raskatov J. A. Chirality Dependence of Amyloid β Cellular Uptake and a New Mechanistic Perspective. ChemBioChem 2019, 20, 1023–1026. 10.1002/cbic.201800708. 30550626 PMC6517241

[ref44] Hu X.; Crick S. L.; Bu G.; Frieden C.; Pappu R. V.; Lee J. M. Amyloid Seeds Formed by Cellular Uptake, Concentration, and Aggregation of the Amyloid-Beta Peptide. Proc. Natl. Acad. Sci. U.S.A. 2009, 106, 20324–20329. 10.1073/pnas.0911281106. 19910533 PMC2787156

[ref45] Gorman P. M.; Yip C. M.; Fraser P. E.; Chakrabartty A. Alternate Aggregation Pathways of the Alzheimer β-Amyloid Peptide: Aβ Association Kinetics at Endosomal PH. J. Mol. Biol. 2003, 325, 743–757. 10.1016/S0022-2836(02)01279-2. 12507477

[ref46] Perez R. G.; Soriano S.; Hayes J. D.; Ostaszewski B.; Xia W.; Selkoe D. J.; Chen X.; Stokin G. B.; Koo E. H. Mutagenesis Identifies New Signals for β-Amyloid Precursor Protein Endocytosis, Turnover, and the Generation of Secreted Fragments, Including Aβ42. J. Biol. Chem. 1999, 274, 18851–18856. 10.1074/jbc.274.27.18851. 10383380

[ref47] Yang W. N.; Ma K. G.; Chen X. L.; Shi L. L.; Bu G.; Hu X. D.; Han H.; Liu Y.; Qian Y. H. Mitogen-Activated Protein Kinase Signaling Pathways Are Involved in Regulating A7 Nicotinic Acetylcholine Receptor-Mediated Amyloid-β Uptake in SH-SY5Y Cells. Neuroscience 2014, 278, 276–290. 10.1016/j.neuroscience.2014.08.013. 25168732

[ref48] Esbjörner E. K.; Chan F.; Rees E.; Erdelyi M.; Luheshi L. M.; Bertoncini C. W.; Kaminski C. F.; Dobson C. M.; Kaminski Schierle G. S. Direct Observations of Amyloid β Self-Assembly in Live Cells Provide Insights into Differences in the Kinetics of Aβ(1-40) and Aβ(1-42) Aggregation. Chem. Biol. 2014, 21, 732–742. 10.1016/j.chembiol.2014.03.014. 24856820 PMC4067742

[ref49] Wesén E.; Jeffries G. D. M.; Dzebo M. M.; Esbjörner E. K. Endocytic Uptake of Monomeric Amyloid-β Peptides Is Clathrin- A Nd Dynamin-Independent and Results in Selective Accumulation of Aβ(1-42) Compared to Aβ(1-40). Sci. Rep. 2017, 7, 2021 10.1038/s41598-017-02227-9. 28515429 PMC5435687

[ref50] Zhang S.; Guaglianone G.; Morris M. A.; Yoo S.; Howitz W. J.; Xing L.; Zheng J. G.; Jusuf H.; Huizar G.; Lin J.; Kreutzer A. G.; Nowick J. S. Expression of N-Terminal Cysteine Aβ42and Conjugation to Generate Fluorescent and Biotinylated Aβ42. Biochemistry 2021, 60, 1191–1200. 10.1021/acs.biochem.1c00105. 33793198 PMC9059633

[ref51] Jin S.; Kedia N.; Illes-Toth E.; Haralampiev I.; Prisner S.; Herrmann A.; Wanker E. E.; Bieschke J. Amyloid-β(1- 42) Aggregation Initiates Its Cellular Uptake and Cytotoxicity. J. Biol. Chem. 2016, 291, 19590–19606. 10.1074/jbc.M115.691840. 27458018 PMC5016693

[ref52] Jameson D. M. Introduction to Fluorescence, 1st ed.; CRC Press, 2014, 10.1201/b16502.

[ref53] Wallrabe H.; Periasamy A. Imaging Protein Molecules Using FRET and FLIM Microscopy. Curr. Opin. Biotechnol. 2005, 16, 19–27. 10.1016/j.copbio.2004.12.002. 15722011

[ref54] Levitt J. A.; Matthews D. R.; Ameer-Beg S. M.; Suhling K. Fluorescence Lifetime and Polarization-Resolved Imaging in Cell Biology. Curr. Opin. Biotechnol. 2009, 20, 28–36. 10.1016/j.copbio.2009.01.004. 19268568

[ref55] Digman M. A.; Caiolfa V. R.; Zamai M.; Gratton E. The Phasor Approach to Fluorescence Lifetime Imaging Analysis. Biophys. J. 2008, 94, L14–L16. 10.1529/biophysj.107.120154. 17981902 PMC2157251

[ref56] Jameson D. M.; Gratton E.; Hall R. D. The Measurement and Analysis of Heterogeneous Emissions by Multifrequency Phase and Modulation Fluorometry. Appl. Spectrosc. Rev. 1984, 20, 55–106. 10.1080/05704928408081716. 6378065

[ref57] Clayton A. H. A.; Hanley Q. S.; Verveer P. J. Graphical Representation and Multicomponent Analysis of Single-Frequency Fluorescence Lifetime Imaging Microscopy Data. J. Microsc. 2004, 213, 1–5. 10.1111/j.1365-2818.2004.01265.x. 14678506

[ref58] Malacrida L.; Ranjit S.; Jameson D. M.; Gratton E. The Phasor Plot: A Universal Circle to Advance Fluorescence Lifetime Analysis and Interpretation. Annu. Rev. Biophys. 2021, 50, 575–593. 10.1146/annurev-biophys-062920-063631. 33957055

[ref59] Giral H.; Lanzano L.; Caldas Y.; Blaine J.; Verlander J. W.; Lei T.; Gratton E.; Levi M. Role of PDZK1 Protein in Apical Membrane Expression of Renal Sodium-Coupled Phosphate Transporters. J. Biol. Chem. 2011, 286, 15032–15042. 10.1074/jbc.M110.199752. 21388960 PMC3083164

[ref60] Hinde E.; Cardarelli F.; Digman M. A.; Gratton E. Changes in Chromatin Compaction during the Cell Cycle Revealed by Micrometer-Scale Measurement of Molecular Flow in the Nucleus. Biophys. J. 2012, 102, 691–697. 10.1016/j.bpj.2011.11.4026. 22325293 PMC3274830

[ref61] Bowers and coworkers observed assemblies of hexamers and dodecamers by nIM-MS and suggest these species to be central building blocks to the assembly of Aβ.

