# Supplementary material for: Elucidating the Oligomerization and Cellular Interactions of a Trimer Derived from Aβ through Fluorescence and Mass Spectrometric Studies
Source: ACS Chem Neurosci. 2022 Jul 27;13(16):2473–82. doi: 10.1021/acschemneuro.2c00313 (PMC9389591; doi:10.1021/acschemneuro.2c00313)

Supporting information for:

Elucidating the Oligomerization and Cellular Interactions of a Trimer Derived from Aβ Through Fluorescence and Mass Spectrometric Studies

Gretchen Guaglianone,[a] Belén Torrado,[c] Yu-Fu Lin,[d,e] Matthew C. Watkins,[a] Vicki H. Wysocki,[d,e] Enrico Gratton,[c] and James S. Nowick*,[a,b]

[a]Department of Chemistry, University of California, Irvine,
Irvine, California 92697, United States
[b]Department of Pharmaceutical Sciences, University of California, Irvine,
Irvine, California 92697, United States
[c]Laboratory for Fluorescence Dynamics, Biomedical Engineering, University of California, Irvine, CA 92697, United States
[d]Resource for Native MS Guided Structural Biology, The Ohio State University, Columbus, OH, 43210, United States
[e]Department of Chemistry and Biochemistry, The Ohio State University, Columbus, OH, 43210, United States

Table of Contents

Figure S1. SDS-PAGE of a mixture of 2AT-L-sCy3 and 2AT-L-sCy5 S3

Figure S2. Micrograph of 2AT-L-sCy3 with SH-SY5Y cells S4

Figure S3. Micrograph of 2AT-L-sCy5 with SH-SY5Y cells S5

Figure S4. Micrograph of concentration gradient of 2AT-L-sCy5 with SH-SY5Y cells S6

Figure S5. Micrograph of 2AT-L-sCy3 with Lysotracker Green S7

Figure S6. Fractional intensity of quenched donor (D_q) S8

Figure S7. Representative clouds on phasor plot S9

General Information S10

Synthesis of Macrocyclic Peptides S11

Synthesis of Fluorescently Labeled 2AT-L S11

SDS-PAGE S12

Steady-state FRET Experiments S12

Native Mass Spectrometry S13

Cell Culture S13

Fluorescence Microscopy S13

FLIM-FRET S14

References and Notes S18
Characterization Data
Characterization of 2AM-L S20
Characterization of 2AM-L_{CC} S24
Characterization of 2AT-L S28
Characterization of 2AT-L-sCy3 S33
Characterization of 2AT-L-sCy5 S38

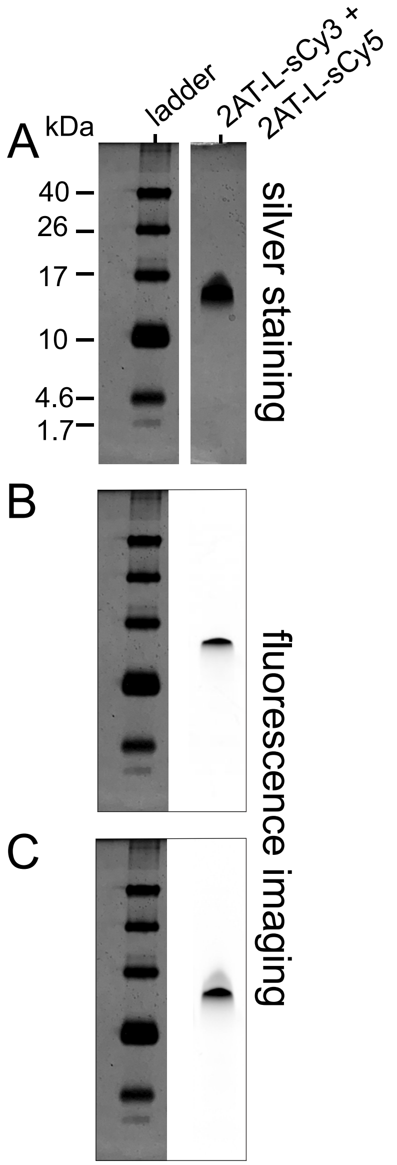

Figure S1. SDS-PAGE of a mixture of trimer 2AT-L-sCy3 and 2AT-L-sCy5. **(A)** Silver-stained image. **(B)** Fluorescence image in the Cy3 channel. **(C)** Fluorescence image in the Cy5 channel. SDS-PAGE was performed in Tris buffer at pH 6.8 with 2% (w/v) SDS on a 16% polyacrylamide gel with 25 μM solution of 2AT-L-sCy3 and 25 μM solution 2AT-L-sCy5 mixed and added to the gel lane. Fluorescence imaging was performed before silver staining.

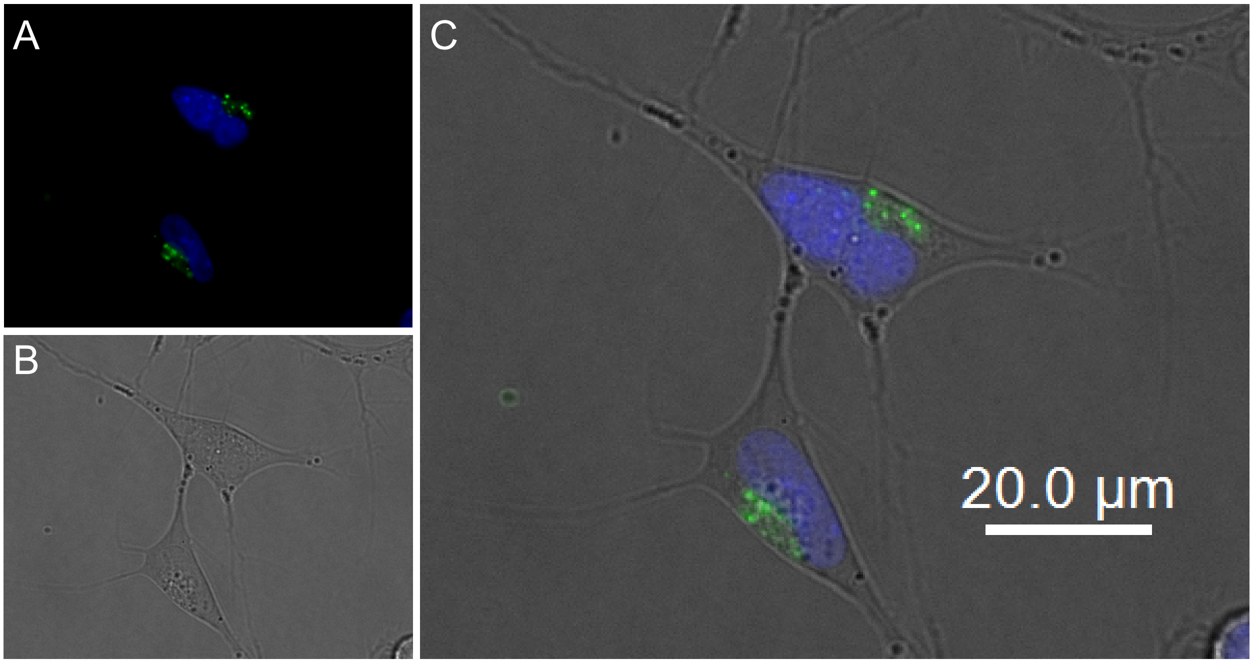

Figure S2. Micrographs illustrating intracellular localization of 2AT-L-sCy3 in SH-SY5Y cells. **(A)** 2AT-L-sCy3 (green) and Hoechst 33342 nuclear stain (blue). **(B)** Brightfield image. **(C)** Merged fluorescent image with brightfield image. Cells were incubated with 5 µM 2AT-L-sCy3 for 6 h at 37°C, counterstained with Hoechst 33342 nuclear stain (blue), and imaged.

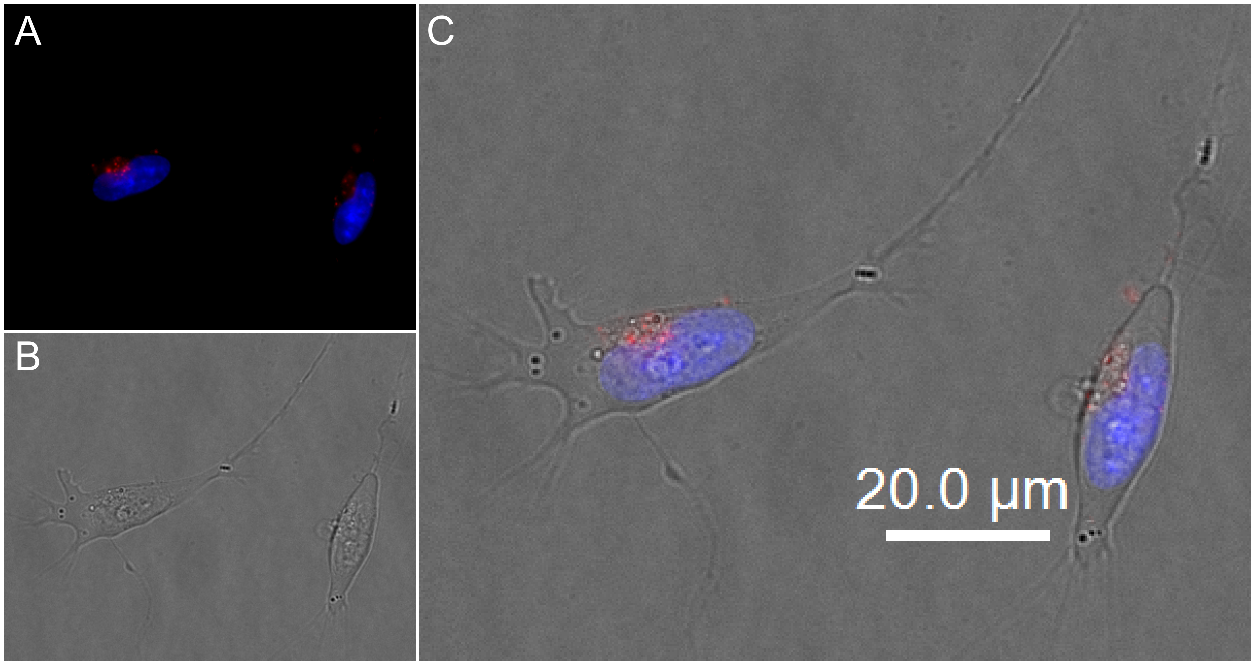

Figure S3. Micrographs illustrating intracellular localization of 2AT-L-sCy5 in SH-SY5Y cells. **(A)** 2AT-L-sCy5 (red) and Hoechst 33342 nuclear stain (blue). **(B)** Brightfield image. **(C)** Merged fluorescent image with brightfield image. Cells were incubated with 5 µM 2AT-L-sCy5 for 6 h at 37°C, counterstained with Hoechst 33342 nuclear stain (blue), and imaged.

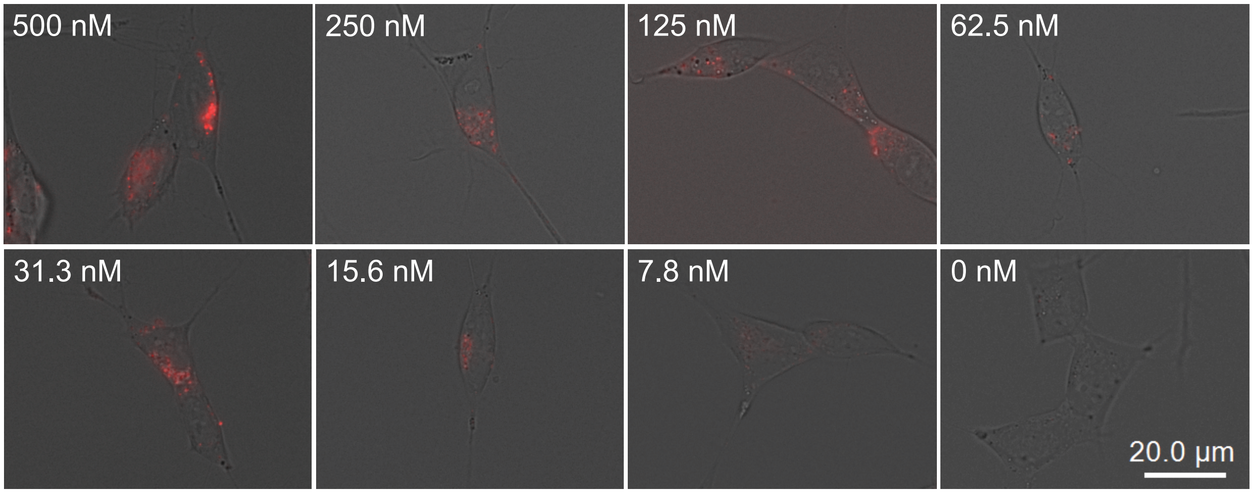

Figure S4. Merged fluorescent and brightfield micrographs of SH-SY5Y cells treated with 7.8–500 nM 2AT-L-sCy3. Cells were incubated with 2AT-L-sCy3 overnight at 37°C and imaged.

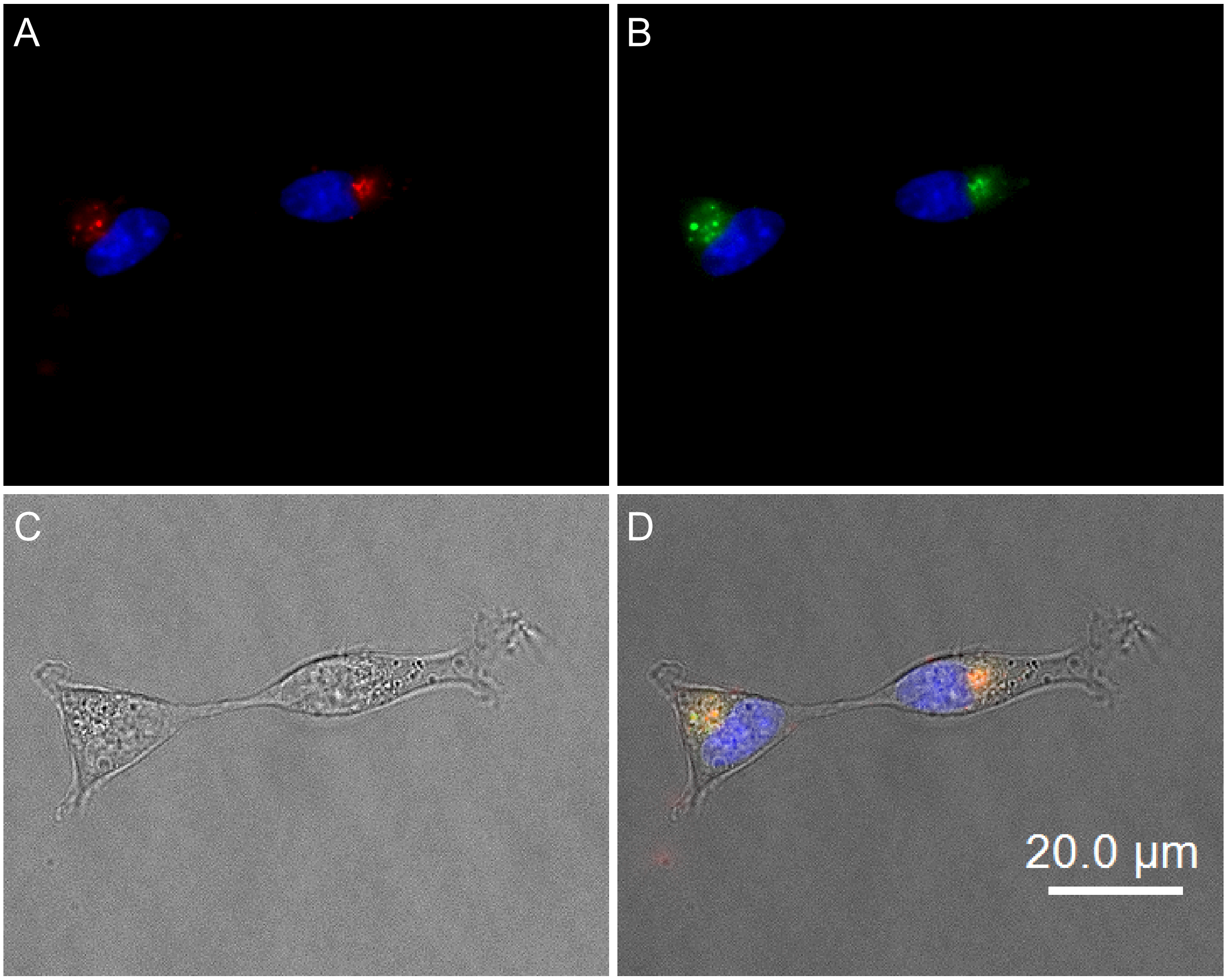

Figure S5. Micrographs illustrating intracellular co-localization of 2AT-L-sCy3 and Lysotracker Green in SH-SY5Y cells. **(A)** 2AT-L-sCy3 (red). **(B)** Lysotracker Green (green). **(C)** Brightfield cell image. **(D)** Merged fluorescent images with brightfield image. Cells were incubated with 5 μM 2AT-L-sCy3 and 100 nM Lysotracker Green for 6 h at 37°C, counterstained with Hoechst 33342 nuclear stain (blue), and imaged.

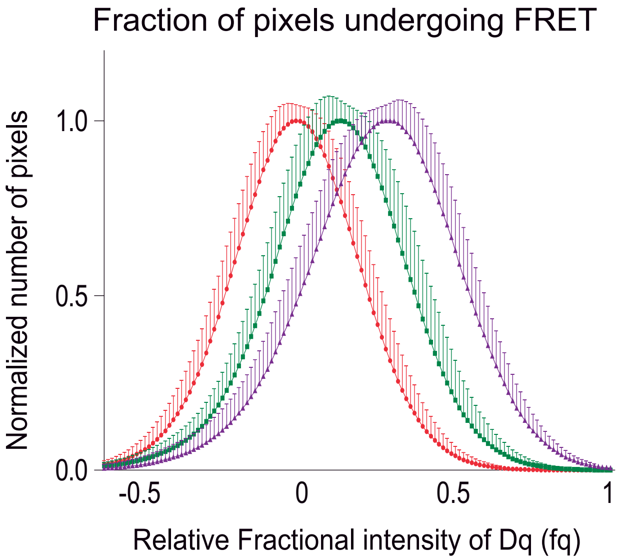

Figure S6. Fractional intensity of D_q (f_q) in all conditions. Fractional intensity is related to the molar fraction and the quantum yield. A shift to increasing values along the x axis is representative of an increase of the fraction of donor undergoing FRET.

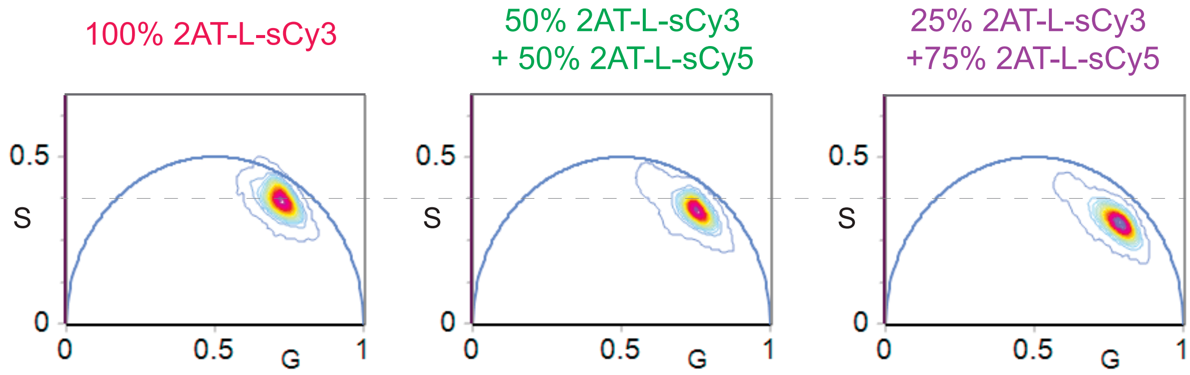

Figure S7. Phasor plot of every pixel from all images in each set of conditions creating a cloud. The red color indicates an increased density of pixels and blue-shifted colors indicate fewer pixels in that position on the phasor plot. Clouds between conditions shift, indicating FRET.

General Information[1]

All Fmoc-protected amino acids including the unnatural amino acid, Boc-ornithine(Fmoc)-OH, were purchased from Chem-Impex or Anaspec. 2-Chlorotrityl chloride resin was purchased from Chem-Impex. Trifluoroacetic acid (TFA), and HPLC grade acetonitrile (MeCN) were purchased from Fischer Scientific. Water was purified to 18 MΩ with a ThermoFisher GenPure Pro water purification system. All other solvents and chemicals were purchased from Alfa Aesar and Sigma Aldrich. All amino acids, resins, solvents, and chemicals were used as received, with the exception that dichloromethane (DCM) and *N,N*-dimethylformamide (DMF) were dried by passage through dry alumina under argon. Analytical HPLC chromatograms were obtained using an Agilent 1260 Infinity II HPLC equipped with Phenomenex bioZen C18 column (150 mm × 4.6 mm, 2.6 µm particle size). HPLC grade acetonitrile (ACN) and 18 MΩ deionized water, each containing 0.1% trifluoroacetic acid, were used as the mobile phase running at 1 mL/min flow rate. Peaks for peptides without fluorophores were detected at 214 nm. Sulfo-cyanine3 labeled peptides were detected at 548 nm and sulfo-cyanine5 labeled peptides were detected at 646 nm in addition to 214 nm. All peptides were monitored using the provided HPLC OpenLAB software.

Semi-preparative scale purification of fluorescently-labeled peptides was done using an Agilent Zorbax 300SB-C18 semi-preparative column (9.4 mm x 250 mm, 5 µm particle size) with a ZORBAX 300SB-C3 preparative guard column (9.4 x 15 mm) on a Rainin Dynamax HPLC with a flow rate of 5.0 mL/min. The C18 column and the guard column were heated to 60 °C in a water bath. Elution was monitored at 214 nm with the accompanying DA Rainin HPLC software. Liquid chromatography-mass spectrometry was performed using a Waters Xevo XS UPLC-QTOF. Spectra were analyzed using the accompanying Waters MassLynx software.

Synthesis of the Macrocyclic Peptide 2AM-L and 2AT-L

Synthesis of peptide 2AM-L and 2AT-L followed the previously outlined procedure of 4AM-L and 4AT-L.[2]

Synthesis of fluorescently-labeled 2AT-L

2AT-L is labeled by treatment with 0.05 molar equivalents of sulfo-cyanine3 (sCy3) or sulfo-cyanine5 (sCy5) NHS esters as follows: 5 mg of NHS-ester sulfo-cyanine3 and NHS-ester sulfo-cyanine5 were purchased from Lumiprobe. The powder was dissolved in water and was transferred into Eppendorf in 17 μg aliquots of fluorophore. The tubes were immediately frozen and then lyophilized. A 75 mM sodium carbonate buffer solution was prepared gravimetrically and the pH was adjusted to 9.6. A 10 mg/mL solution of 2AT-L was prepared gravimetrically by dissolving the lyophilized trimer in the appropriate amount of 18 MΩ deionized water. 300 μL of 10 mg/mL stock solution was added to a fresh Eppendorf tube. 700 μL of carbonate buffer solution was added to the Eppendorf tube for a total volume of 1 mL. Lyophilized fluorophore was added to the reaction mixture by transferring 100 μL of solution into one aliquot of lyophilized fluorophore. The fluorophore was dissolved and the solution was transferred back to the larger reaction mixture. The reaction mixture was protected from light with black felt and rocked gently for 1 h. After 1 h, the reaction mixture was directly injected onto an RP-HPLC.

Semi-preparative scale purification of the fluorescently-labeled peptides was done using an Agilent Zorbax 300SB-C18 semi-preparative column (9.4 mm x 250 mm, 5 μm particle size) with a ZORBAX 300SB-C3 preparative guard column (9.4 x 15 mm) on a Rainin Dynamax HPLC with a flow rate of 5.0 mL/min. The C18 column and the guard column were heated to 60 °C in a water bath and were eluted over a gradient of 20–45% MeCN over 80 min. Elution was monitored at 214 nm with the accompanying DA Rainin HPLC software. Liquid chromatography-mass spectrometry was performed using a Waters Xevo XS UPLC-QTOF. Spectra were analyzed using the accompanying Waters MassLynx software. Pure fractions were concentrated by rotary evaporation and lyophilized. The purified labeled trimers were characterized by analytical HPLC and liquid chromatography-mass spectrometry. The fractions containing unlabeled 2AT-L were concentrated for subsequent use.

Stock solutions of fluorescently-labeled analogues were prepared spectrophotometrically. Lyophilized peptide was dissolved in 18 MΩ deionized water and the absorbance was measured

using the λ_{max} of the respective fluorophore (2AT-L-sCy3 λ_{max}= 548 nm, 2AT-L-sCy5 λ_{max}= 646 nm). The published molar extinction coefficients were used to calculate sample concentration. A typical synthesis yielded approximately 150 µL of 1 mg/mL solution. [Labeling 3 mg of trimer by this procedure typically permits isolation of ca. 150 µg of singly-labeled trimer 2AT-L-sCy3 or 2AT-L-sCy5 as the TFA salt.]

SDS-PAGE[3]

Solutions of the unlabeled peptides were prepared gravimetrically by dissolving the lyophilized peptide in the appropriate amount of 18 MΩ deionized water to achieve a 10 mg/mL stock. Stock solutions of the fluorescently labeled peptides were prepared and concentrations determined spectrophotometrically as described above. Stock solutions of all peptides were diluted with 18 MΩ deionized water to create 50 µM sample solutions.1 µL of 6X SDS-PAGE sample loading buffer (G Biosciences) was added per 5 µL sample solution to create working solutions. A 5 µL aliquot of each working solution was run on a 16% polyacrylamide gel with a 4% stacking polyacrylamide gel. The gel was run at a constant 60 volts for approximately 4 h. Reagents and gels for Tricine SDS-PAGE were prepared according to recipes and procedures detailed in Schägger, H. *Nat. Protoc.* **2006**, *1*, 16–22.[4]

Prior to silver staining, a fluorescence image of the gel was obtained using a Gel Doc XR+ Gel Documentation System (BioRad) in the Cy3 and Cy5 channels. Staining with silver nitrate was then used to visualize the peptides in the gel. Reagents for silver staining were prepared according to procedures detailed in Simpson, R. J. *Cold Spring Harb. Protoc.* **2007**.[5] The gel was then silver stained following the procedures previously described.[3]

Steady-state FRET experiments

Freshly prepared stock solutions of fluorescently labeled trimer analogues were used to prepare samples. Each sample ratio was prepared immediately prior to analysis. Samples of 2AT-L-sCy3 and 2AT-L-sCy5 were prepared to a final volume of 150 µL at a total concentration of 5 µM in the following ratios: 100:0, 80:20, 60:40, 50:50, 40:60, 20:80, 0:100. Samples were prepared in Eppendorf tubes and transferred to a 10 mm quartz sub-micro fluorometer cell (Starna) for analysis. An Agilent Cary Eclipse Fluorescence Spectrophotometer was used to

excite trimer mixtures at 490 nm record emission spectra. Emission was collected from 555 nm to 750 nm with a slit width of 5 nm. Sample emission at 662 nm (maximum emission of sCy5) was selected to graph emission output.

Native IM-MS

Solution of unlabeled trimer was prepared gravimetrically by dissolving the lyophilized peptide in the appropriate amount of 18 MΩ deionized water to achieve a 10 mg/mL stock. Stock solutions of the fluorescently labeled peptides were prepared and concentrations determined spectrophotometrically as described previously. The stock solutions were diluted to 40 μM in 400 mM ammonium acetate immediately before each mass spectrometric experiment. A Waters SELECT SERIES Cyclic Ion Mobility Spectrometry System[6] (cIMS) was used to conduct mass spectrometry analysis. Samples were ionized using nanoelectrospray ionization (nano-ESI). Nano-ESI glass capillary tips were pulled in-house using a Sutter Instruments P-97 micropipette tip puller (Novato, CA). When performing experiments on the cIMS, the cIMS was tuned as follows: capillary voltage, 0.4 to 0.6 kV; cone voltage, 20 V; source temperature, 25 °C; trap CE, 4 V; transfer CE, 4 V; trap and transfer gas, N_2 at 7.0 mL/min; IMS pressure, 1.8 mbar; TW static height, 22 V; TW velocity, 375 m/s; pushes per bin, 2.

Cell Culture

SH-SY5Y neuroblastoma cell cultures (ATCC® CRL-2266™) were maintained in 1:1 mixture of Dubelcco's modified Eagle medium and Ham's F12 (DMEM:F12) medium supplemented with 10% heat-inactivated fetal bovine serum (FBS), 100 U/mL penicillin and 100 μg/mL streptomycin at pH 7.4 in a humidified 5% CO_2 atmosphere at 37 °C using a Fischer Scientific Forma Series 3 Water Jacketed CO_2 Incubator. All experiments were performed using ca. 60–80% confluent cells on passages ranging from 2–10.

Fluorescence Microscopy[7]

Cell Preparation, Treatment, and Imaging. SH-SY5Y cells were plated in an Ibidi μ-Slide 8 Well Chamber Slide (Ibidi catalog number 80826) at 80,000 cells per well. Cells were incubated in 500 μL of a 1:1 mixture of DMEM:F12 media supplemented with 10% fetal bovine serum, 100 U/mL penicillin, and 100 μg/mL streptomycin at 37 °C in a 5% CO_2 atmosphere and

allowed to adhere to the bottom of the slide for 48 hours. A solution of 100 nM of lysotracker-containing media was prepared by adding Lysotracker Green DND-26 (ThermoFischer) into serum-free, phenol red-free 1:1 DMEM/F12 media. Solutions of 2AT-L-sCy3, 2AT-L-sCy5, or both were prepared to a final volume of 200 µL and a final concentration of 5 µM in either serum-free, phenol red-free 1:1 DMEM/F12 media or serum-free, phenol red-free 1:1 DMEM/F12 media with Lysotracker Green DND-26.

After cells were incubated for 48 hours, the media was removed and replaced with solutions with fluorescently labeled analogues with and without Lysotracker Green DND-26. Control wells were treated with media containing no peptide. The slide was incubated in the microscope chamber for 6 hours. media was removed from the well slide containing the cells and replaced with 200 µL of 1 µg/mL Hoechst 33342 in serum-free, phenol red-free 1:1 DMEM:F12 media. After 30 minutes, the Hoechst-containing media was removed and replaced with 200 µL of serum-free, phenol red-free 1:1 DMEM:F12 media. The cells were imaged using a Keyence BZ-X810 fluorescence microscope. Images were collected with a 60x oil immersion objective lens. Micrographs of treated cells were recorded using the DAPI filter cube [excitation wavelength = 350/50 nm (325–375 nm) and emission wavelength = 460/50 nm (435–485nm)] for Hoechst nuclear marker, the Cy3 filter cube [excitation wavelength = 545/25 nm (532.5–557.5 nm) and emission wavelength = 605/70 nm (570–640 nm)] for 2AT-L-sCy3, the Cy5 filter cube [excitation wavelength = 620/60 nm (590–650 nm) and emission wavelength = 700/75 nm (662.5–737.5 nm)] for 2AT-L-sCy5, and the GFP filter cube [excitation wavelength = 470/40 nm (450–490 nm) and emission wavelength = 525/50 nm (500–550 nm)] for Lysotracker Green DND-26 (ThermoFischer).The image brightness of the channels was adjusted using BZ-X810 Analyzer software.

FLIM-FRET

Sample Preparation and Imaging. SH-SY5Y cells were plated in an Ibidi µ-Slide 8 Well Chamber Slide (Ibidi catalog number 80826) at 80,000 cells per well. Cells were incubated in 500 µL of a 1:1 mixture of DMEM:F12 media supplemented with 10% fetal bovine serum, 100 U/mL penicillin, and 100 µg/mL streptomycin at 37 °C in a 5% CO_2 atmosphere and allowed to adhere to the bottom of the slide for 48 hours. After 48 h, media was removed from the Ibidi µ-Slide 8 Well Chamber Slide and replaced with 200 µL of 5 µM fluorescently labeled trimer

solutions prepared in serum-free, phenol red-free DMEM:F12. Cells were incubated with peptide solutions for 6 h. Cells were incubated with the following ratios of 2AT-L-sCy3 and 2AT-L-sCy5: 100:0 (5 μM:0), 50:50 (2.5 μM: 2.5 μM), and 25:75 (1.25 μM: 3.75 μM). After 6 h, the media was removed and replaced with 200 μL serum-free, phenol red-free DMEM:F12 media and the cells were imaged.

All FLIM-FRET data was acquired using a two-channel ISS-ALBA5 confocal microscope equipped with a white laser source and acusto-optic tunable filter (NKT SuperK EXTREME, SuperK SELECT), and a digital frequency domain setup the ISS A320 FastFLIM acquisition unit for lifetime measurement. The repetition frequency of the laser is 78 MHz. A 60x oil immersion objective of 1.2 NA (Olympus) was used for all experiments. Intensity and lifetime data were simultaneously acquired by raster-scanning the one-photon laser. sCy3 was excited at 490 nm laser selected from the white laser, and a 488/25 dichroic mirror was used to separate the fluorescence signal from the laser light. The donor and acceptor signals were split between two avalanche photo-diodes detectors (Excelitas Technologies) using a long-pass 640 nm dichroic mirror. Additionally, bandwidth filters were placed in front of each detector: for 2AT-L-sCy3 a 575/50 (donor channel) and for 2AT-L-sCy5 a 679/41 (acceptor channel). Only the donor channel was used to analyze FLIM-FRET data. The acceptor channel was used only to obtain intensity images to check the presence of sCy5. The pixel frame size was set to 256, pixel size of 0.097 μm, and the pixel dwell time was set to 16 μs/pixel. For each FLIM experiment, 20 frames were integrated. All images accumulated contained from 0 to 2000 photons per pixel. Calibration of the system and phasor plot space was performed by measuring coumarin 6 (in ethanol), which has a known single-exponential lifetime of 2.4 ns.

Data Processing-Phasor Transformation. The FLIM data was collected and processed using the SimFCS software developed at the Laboratory for Fluorescence Dynamics (LFD) (available at https://www.lfd.uci.edu/). The FLIM data was analyzed using the phasor approach.[9–12] The intensity decays collected from each pixel of the image was transformed to the phasor plot using a fast Fourier transformation. The sine and cosine components are plotted against each other in the phasor plot, where the intensity decay from each pixel is transformed to a coordinate value, expressed by G (cosine) and S (sine). The uncertainty and hence the spread of the phasors position depends on the number of counts in each pixel. A background correction of 40 counts/pixel was employed using the intensity histogram. The extent of the autofluorescence

signal was determined was determined by measuring cells without the presence of fluorophores using the same cell preparation as that of the treated samples and was ~ 3% of the total signal.

The G and S value for the phasor position corresponding to every single cell (each FLIM image) was determined from the center of the distribution of the phasor points from that cell using the method described before.[13] The phasor distribution of the of the donor only cells (unquenched, D_{unq}) were determined using cells incubated with 2AT-L-sCy3. The occurrence of FRET was analyzed in the phasor plot by quantifying the phasor shift occurring between the donor only and the donor in the presence of acceptor (D_q). The FRET trajectory for the donor is curved as the donor lifetime is gradually quenched by the FRET process. The shape of the FRET trajectory and deviation from the linear combination between the donor only and donor-acceptor phasor positions is dependent on the contribution from background. The measurement of autofluorescence under the same excitation wavelength is required for the calculation of the trajectory.[14]

Each pixel of an image can contain donor only, donor-acceptor, or a combination of different fractions of donor (f_q) and donor-acceptor (f_{unq}). Hence, the resulting phasor appears along the line joining the phasor positions of the donor and donor-acceptor at a distance from D_{unq} that is proportional to the fraction of interacting donors f_q.[15,16] This can become curved if the donor only and donor-acceptor samples have different amount of fractional intensity contribution from the autofluorescence as donor-acceptor sample can be less fluorescent. In our experiments the donor only and donor-acceptor phasor positions were located along a line, not in the curved trajectory, due to the very small amount of autofluorescence and can be expressed as a simple liner combination of D_q and D_{unq} and not a curved trajectory. The fractional intensity of interacting donors f_q was calculated using linear additive properties of phasor plot.[13,16]

Phasor transformation was performed using SimFCS. The fluorescence lifetime decay curve I(t) in every pixel of a FLIM image is transformed into phasor plot coordinates according to:

$$S = \frac{\int_0^T I(t)\sin(n\omega t)dt}{\int_0^T I(t)dt} \qquad \text{(Eq 1)}$$

$$G = \frac{\int_0^T I(t)\cos(n\omega t)\,dt}{\int_0^T I(t)\,dt} \qquad \text{(Eq 2)}$$

where ω is the laser repetition angular frequency (2πf), the harmonic number n represents the integer number of whole cycles the trigonometric function has in the repetition period. T is the repetition frequency of the laser.

If the decay curve is single exponential $I(t) = Ae^{\frac{-t}{\tau}}$, the Eqs. 1 and 2 can be solved to obtain the coordinates of the phasor by:

$$s = \frac{n\omega\tau}{1+(n\omega\tau)^2} \quad \text{(Eq 3)}$$

$$g = \frac{1}{1+(n\omega\tau)^2} \quad \text{(Eq 4)}$$

Combining Eqs. 3 and 4 gives the expression $s_i^2 = g_i - g_i^2$ where the pure decays (single exponential) lie, that is represented by a semi-circle in the phasor plot, which is called "universal circle". A phasor corresponding to a long lifetime will appear close to the (0,0) point, meanwhile one of short lifetime will appear closer to the point (1,0).

If the decay curve is a combination of exponential curves $I(t) = \sum_1^N A_i e^{\frac{-t}{\tau}}$, the coordinates of the phasor plot are the sum of the phasor transformations of the pure components:

$$\sum_1^N f_i s_i = S \quad \text{(Eq 6)}$$

$$\sum_1^N f_i g_i = G \quad \text{(Eq 7)}$$

Upper case $(S \ , G \ )$ are the coordinates of our data points, lower case $(s_i \ , g_i \ )$ are the unknown coordinates of the pure components and f_i their respective photon fractions. The fractions have to add up to unity $\sum_1^N f_i = 1$.

References and Notes

(1) The chemicals and instruments required for the synthesis of macrocyclic β-sheet peptide 2AM-L and 2AT-L are similar to those used in our laboratory's previous publications. This information was either adapted from or taken verbatim from Kreutzer, A. G.; Yoo, S.; Spencer, R. K.; Nowick, J. S. Stabilization, Assembly, and Toxicity of Trimers Derived from Aβ. *J. Am. Chem. Soc.* **2017**, *139* (2), 966–975. https://doi.org/10.1021/jacs.6b11748.

(2) Haerianardakani, S.; Kreutzer, A. G.; Salveson, P. J.; Samdin, T. D.; Guaglianone, G. E.; Nowick, J. S. Phenylalanine Mutation to Cyclohexylalanine Facilitates Triangular Trimer Formation by β-Hairpins Derived from Aβ. *J. Am. Chem. Soc.* **2020**, *142* (49), 20708–20716. https://doi.org/10.1021/jacs.0c09281.

(3) Peptides were run on SDS-PAGE and silver stained following a protocol similar to those published previously in our laboratory. The procedures were either adapted from or taken verbatim from Kreutzer, A. G.; Yoo, S.; Spencer, R. K.; Nowick, J. S. Stabilization, Assembly, and Toxicity of Trimers Derived from Aβ. *J. Am. Chem. Soc.* **2017**, *139* (2), 966–975. https://doi.org/10.1021/jacs.6b11748.

(4) Schägger, H. Tricine-SDS-PAGE. *Nat. Protoc.* **2006**, *1*, 16–22.

(5) Simpson, R. J. Staining Proteins in Gels with Silver Nitrate. *Cold Spring Harb. Protoc.* **2007** doi: 10.1101/Pdb.Prot4727.

(6) Snyder, D. T.; Jones, B. J.; Lin, Y. F.; Cooper-Shepherd, D. A.; Hewitt, D.; Wildgoose, J.; Brown, J. M.; Langridge, J. I.; Wysocki, V. H. Surface-Induced Dissociation of Protein Complexes on a Cyclic Ion Mobility Spectrometer. *Analyst* **2021**, *146* (22), 6861–6873. https://doi.org/10.1039/d1an01407b.

(7) Fluorescently-labeled 2AT-L analogues were imaged following a protocol similar to those published previously by our laboratory. This information was either adapted from or taken verbatim from Zhang, S.; Guaglianone, G.; Morris, M. A.; Yoo, S.; Howitz, W. J.; Xing, L.; Zheng, J. G.; Jusuf, H.; Huizar, G.; Lin, J.; Kreutzer, A. G.; Nowick, J. S. Expression of N-Terminal Cysteine Aβ42 and Conjugation to Generate Fluorescent and Biotinylated Aβ42. *Biochemistry* **2021**, *60* (15), 1191–1200. https://doi.org/10.1021/acs.biochem.1c00105.

(8) Zhang, S.; Guaglianone, G.; Morris, M. A.; Yoo, S.; Howitz, W. J.; Xing, L.; Zheng, J. G.;

Jusuf, H.; Huizar, G.; Lin, J.; Kreutzer, A. G.; Nowick, J. S. Expression of N-Terminal Cysteine Aβ42and Conjugation to Generate Fluorescent and Biotinylated Aβ42. *Biochemistry* **2021**, *60* (15), 1191–1200. https://doi.org/10.1021/acs.biochem.1c00105.

(9) Digman, M. A.; Caiolfa, V. R.; Zamai, M.; Gratton, E. The Phasor Approach to Fluorescence Lifetime Imaging Analysis. *Biophys. J.* **2008**, *94* (2), 14–16. https://doi.org/10.1529/biophysj.107.120154.

(10) Malacrida, L.; Ranjit, S.; Jameson, D. M.; Gratton, E. The Phasor Plot: A Universal Circle to Advance Fluorescence Lifetime Analysis and Interpretation. *Annu. Rev. Biophys.* **2021**, *50*, 575–593. https://doi.org/10.1146/annurev-biophys-062920-063631.

(11) Jameson, D. M.; Gratton, E.; Hall, R. D. The Measurement and Analysis of Heterogeneous Emissions by Multifrequency Phase and Modulation Fluorometry. *Appl. Spectrosc. Rev.* **1984**, *20* (1), 55–106. https://doi.org/10.1080/05704928408081716.

(12) Clayton, A. H. A.; Hanley, Q. S.; Verveer, P. J. Graphical Representation and Multicomponent Analysis of Single-Frequency Fluorescence Lifetime Imaging Microscopy Data. *J. Microsc.* **2004**, *213* (1), 1–5. https://doi.org/10.1111/j.1365-2818.2004.01265.x.

(13) Ranjit, S.; Malacrida, L.; Jameson, D. M.; Gratton, E. Fit-Free Analysis of Fluorescence Lifetime Imaging Data Using the Phasor Approach. *Nat. Protoc.* **2018**, *13* (9), 1979–2004. https://doi.org/10.1038/s41596-018-0026-5.

(14) Hinde, E.; Cardarelli, F.; Digman, M. A.; Gratton, E. Changes in Chromatin Compaction during the Cell Cycle Revealed by Micrometer-Scale Measurement of Molecular Flow in the Nucleus. *Biophys. J.* **2012**, *102* (3), 691–697. https://doi.org/10.1016/j.bpj.2011.11.4026.

(15) Giral, H.; Lanzano, L.; Caldas, Y.; Blaine, J.; Verlander, J. W.; Lei, T.; Gratton, E.; Levi, M. Role of PDZK1 Protein in Apical Membrane Expression of Renal Sodium-Coupled Phosphate Transporters. *J. Biol. Chem.* **2011**, *286* (17), 15032–15042. https://doi.org/10.1074/jbc.M110.199752.

(16) Torrado, B.; Malacrida, L.; Ranjit, S. Linear Combination Properties of the Phasor Space in Fluorescence Imaging. *Sensors* **2022**, *22* (3). https://doi.org/10.3390/s22030999.

Characterization Data

Characterization of peptide 2AM-L

Analytical HPLC trace

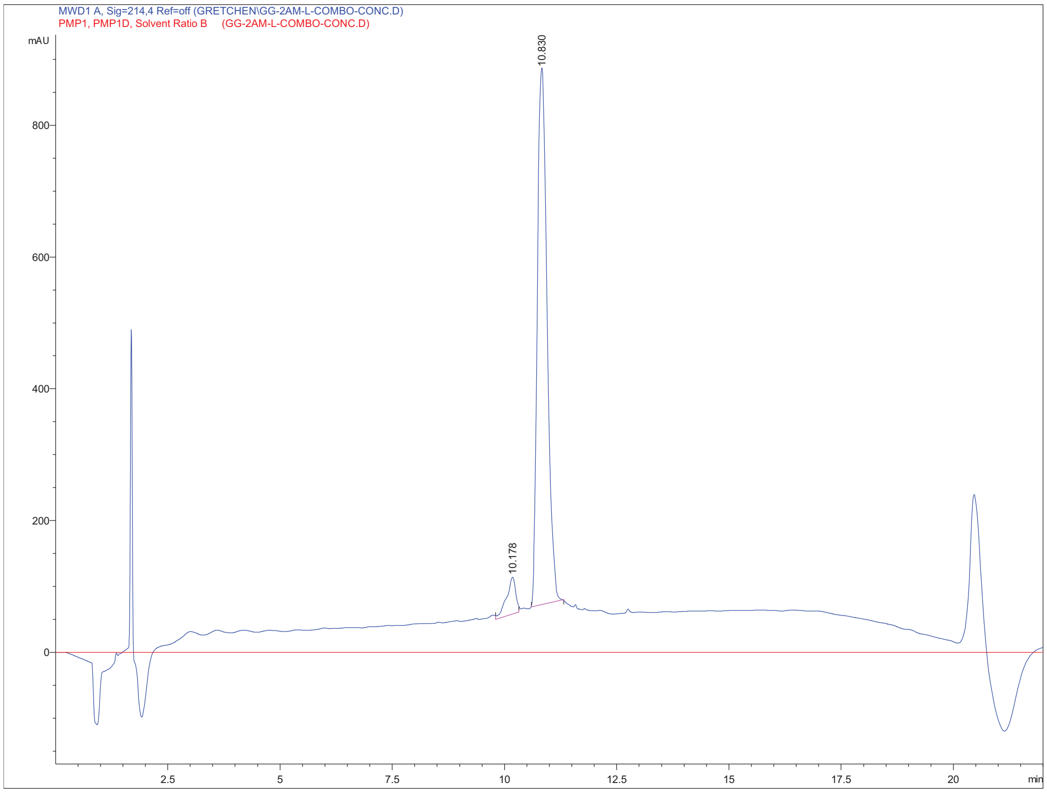

Signal 1: MWD1 A, Sig=214,4 Ref=off

Peak #	RetTime [min]	Type	Width [min]	Area [mAU*s]	Height [mAU]	Area %
1	10.178	MM	0.2374	795.36218	55.83872	6.0593
2	10.830	MM	0.2522	1.23309e4	814.92358	93.9407

Mass spectrum of peptide 2AM-L.

Calculated $[M+H]^+$ of peptide 2AM-L: 2173.24

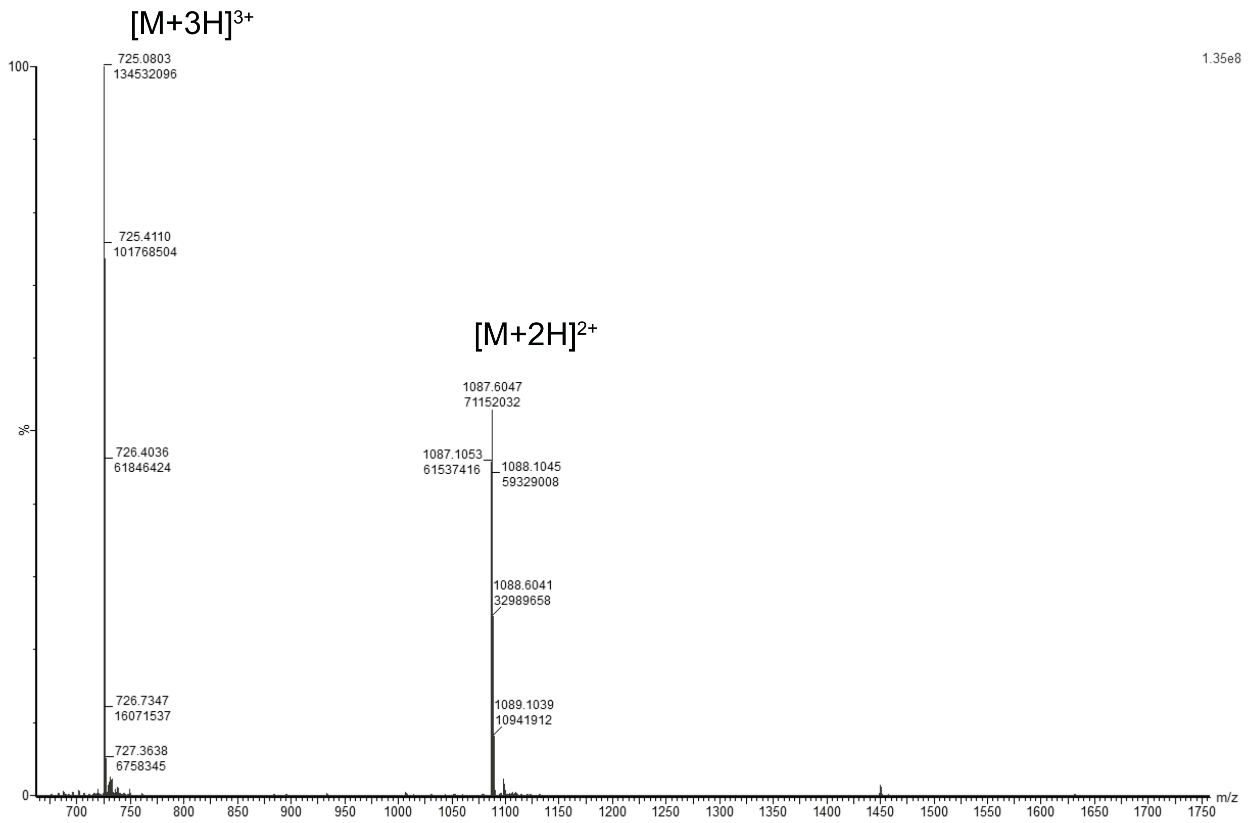

Expansion mass spectrum of peptide 2AM-L.

Calculated $[M+H]^+$ of peptide 2AM-L: 2173.24

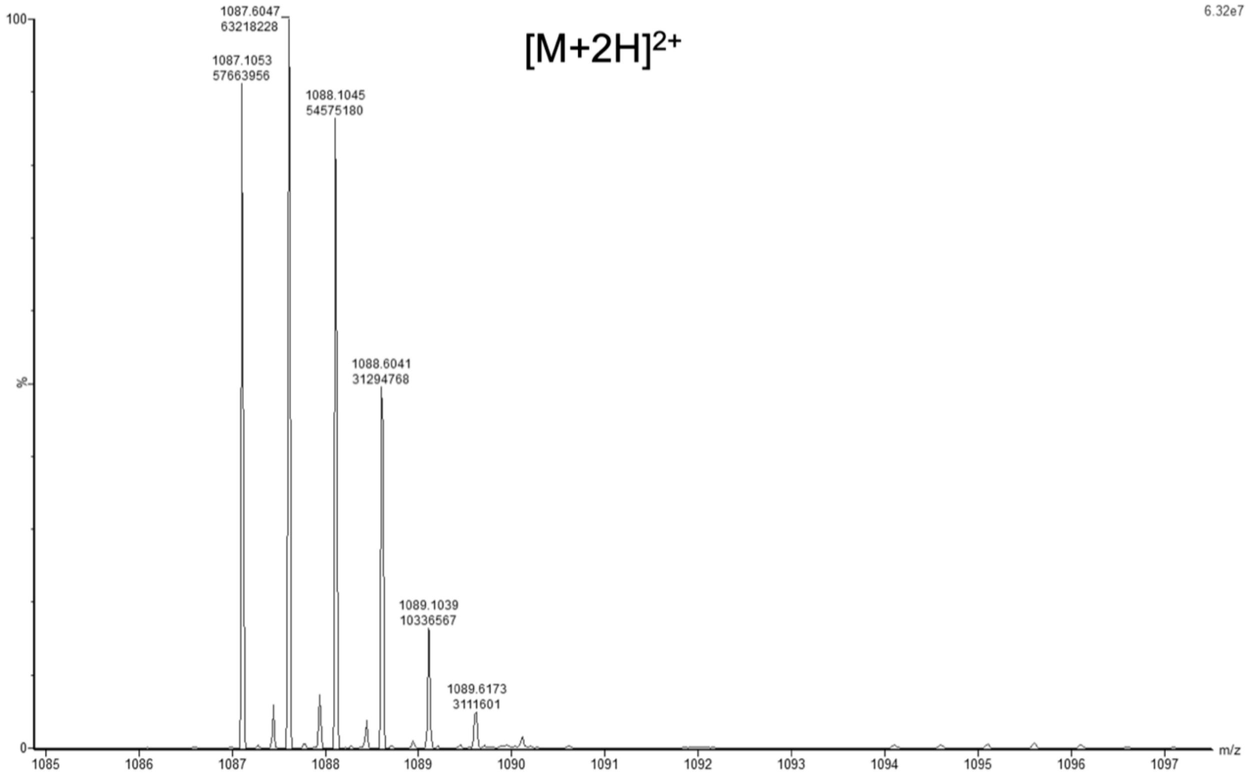

Expansion mass spectrum of peptide 2AM-L.

Calculated $[M+H]^+$ of peptide 2AM-L: 2173.24

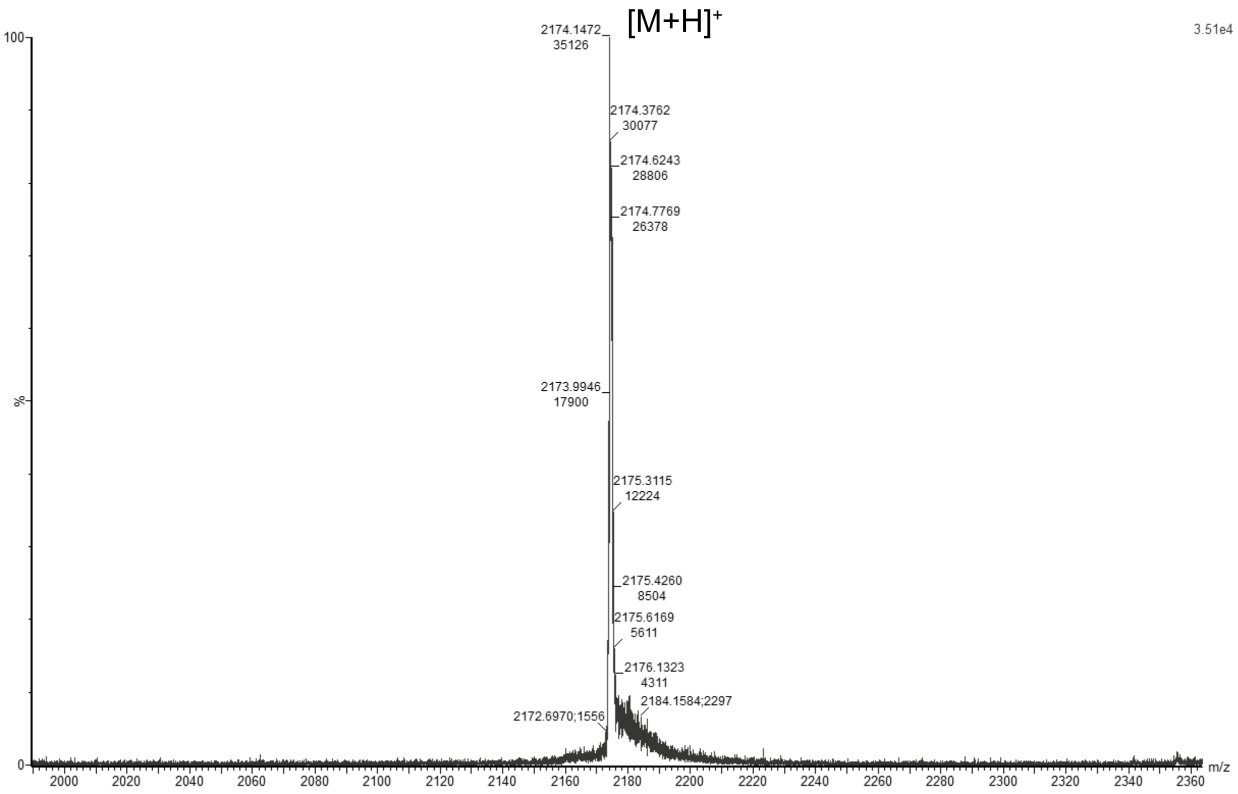

Characterization of peptide 2AM-L_{CC}

Analytical HPLC trace

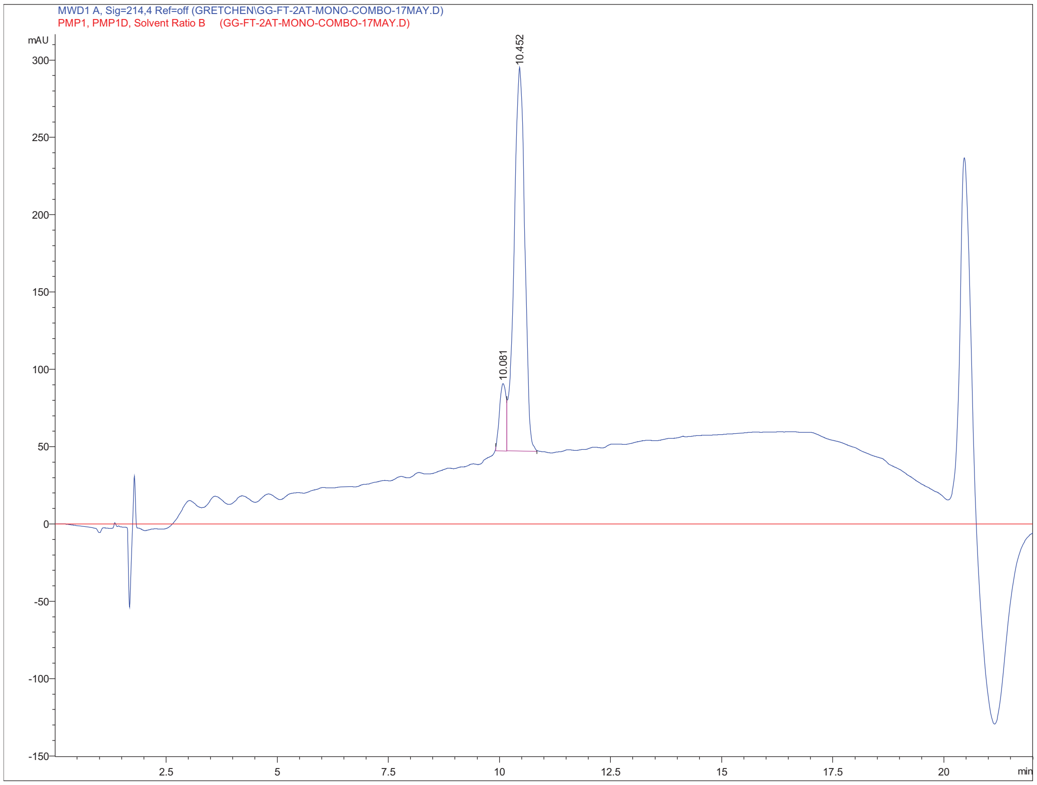

Signal 1: MWD1 A, Sig=214,4 Ref=off

Peak #	RetTime [min]	Type	Width [min]	Area [mAU*s]	Height [mAU]	Area %
1	10.081	MM	0.1710	445.97171	43.47296	9.7557
2	10.452	MM	0.2770	4125.44141	248.21205	90.2443

Mass spectrum of peptide 2AM-L_{CC}.

Calculated $[M+H]^+$ of peptide 2AM-L_{CC}: 2195.14

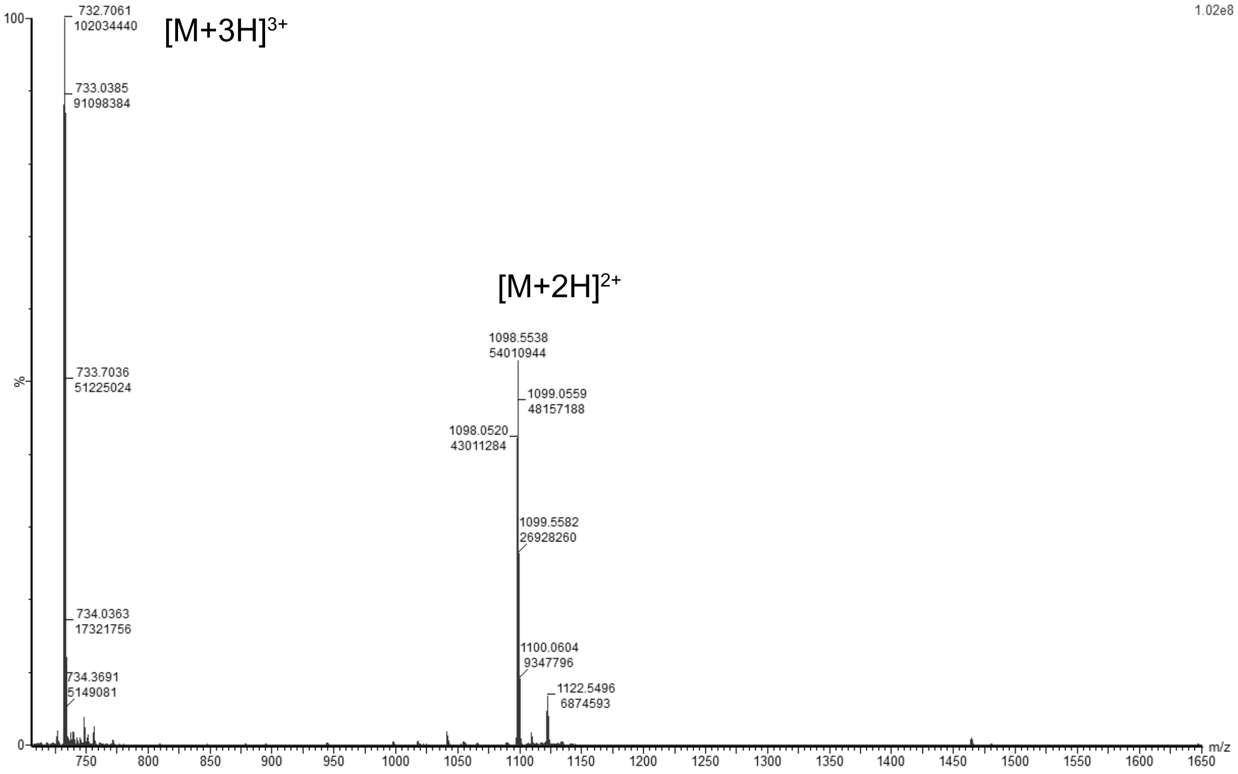

Expansion mass spectrum of peptide 2AM-L_{CC}.

Calculated $[M+H]^+$ of peptide 2AM-L_{CC}: 2195.14

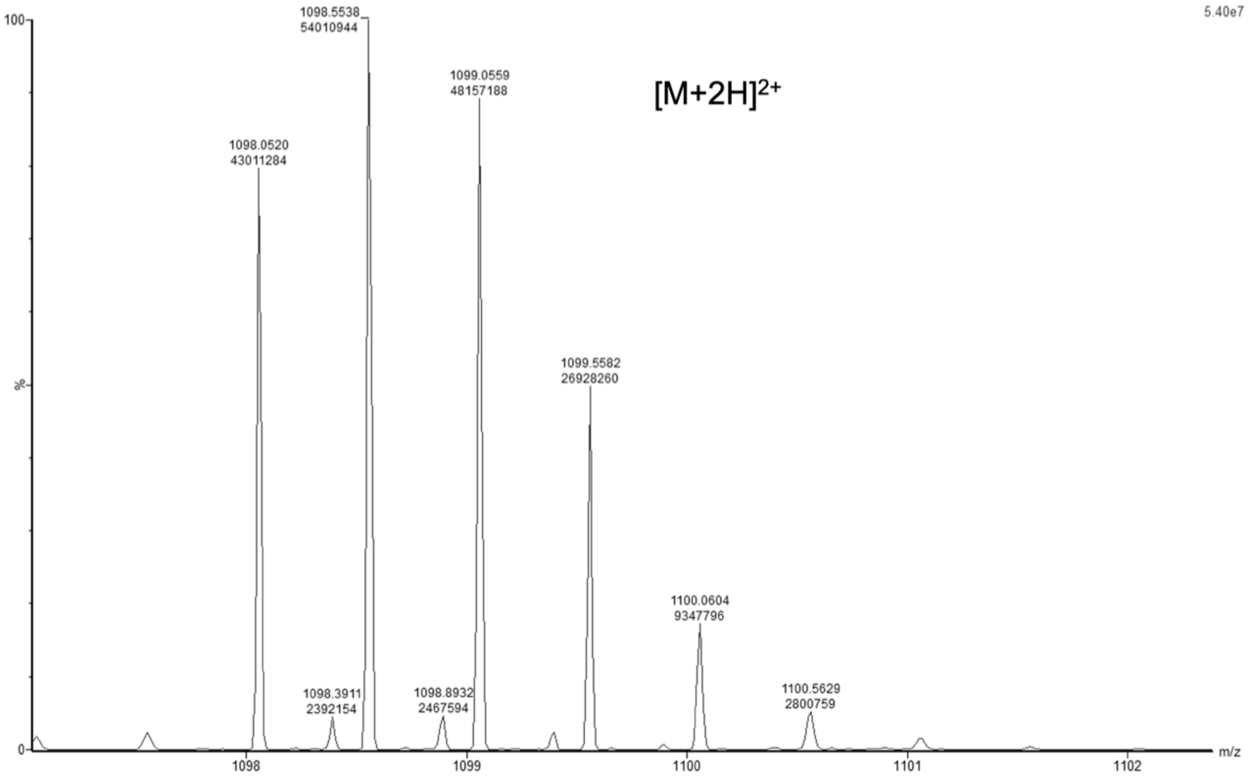

Expansion mass spectrum of peptide 2AM-L_{CC}.

Calculated $[M+H]^+$ of peptide 2AM-L_{CC}: 2195.14

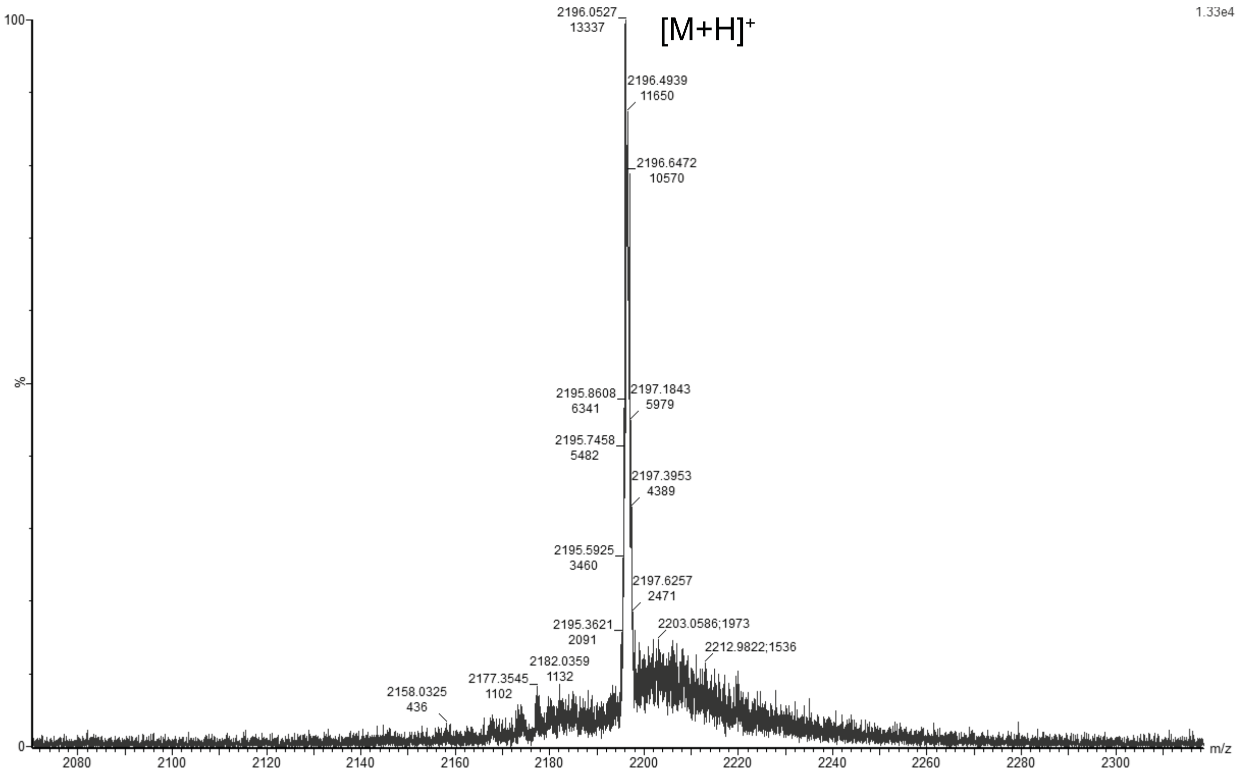

Characterization of trimer 2AT-L

Analytical HPLC trace

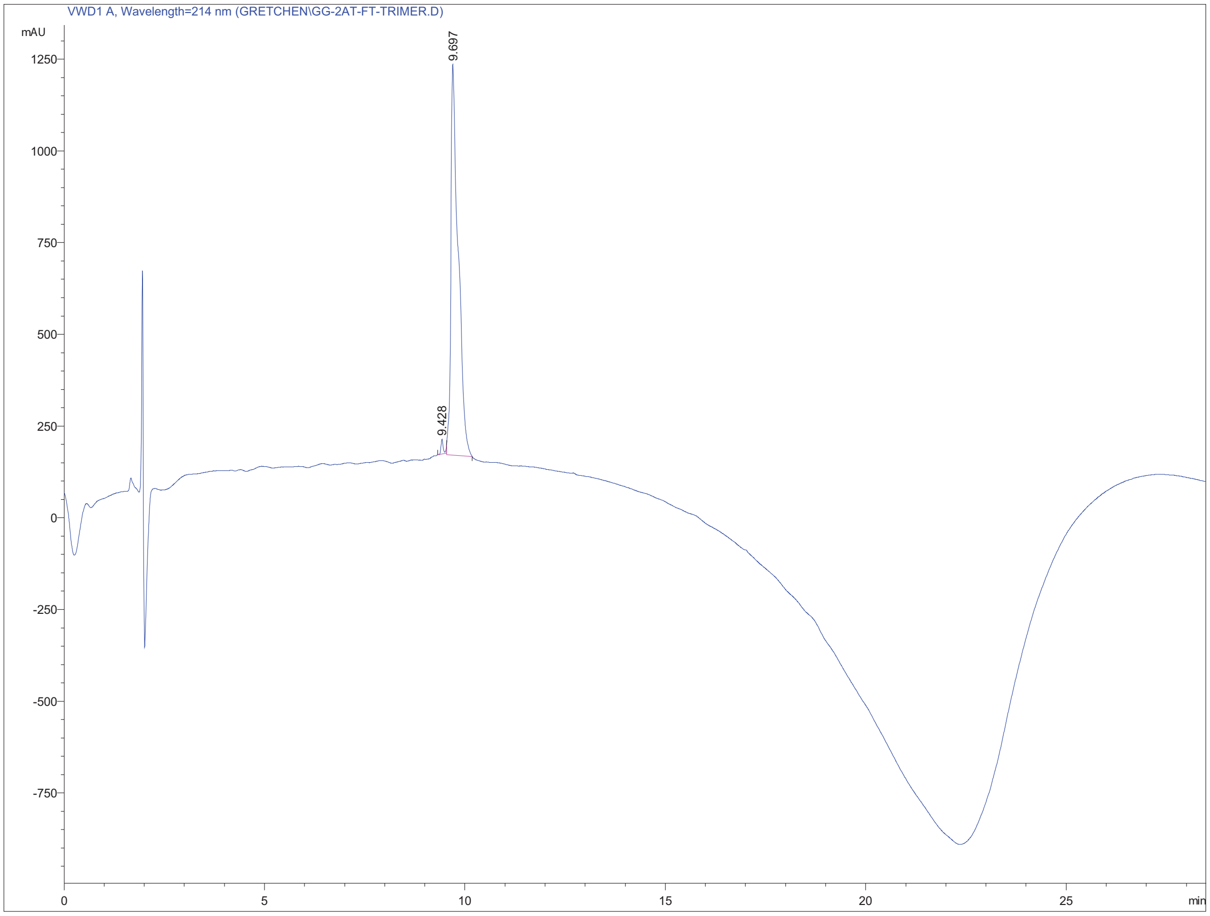

Signal 1: VWD1 A, Wavelength=214 nm

Peak #	RetTime [min]	Type	Width [min]	Area mAU *s	Height [mAU]	Area %
1	9.428	MM	0.0706	171.36655	40.45287	1.2561
2	9.697	MM	0.2106	1.34710e4	1066.05396	98.7439

Mass spectrum of trimer 2AT-L.

Calculated $[M+H]^+$ of trimer 2AT-L: 6577.34

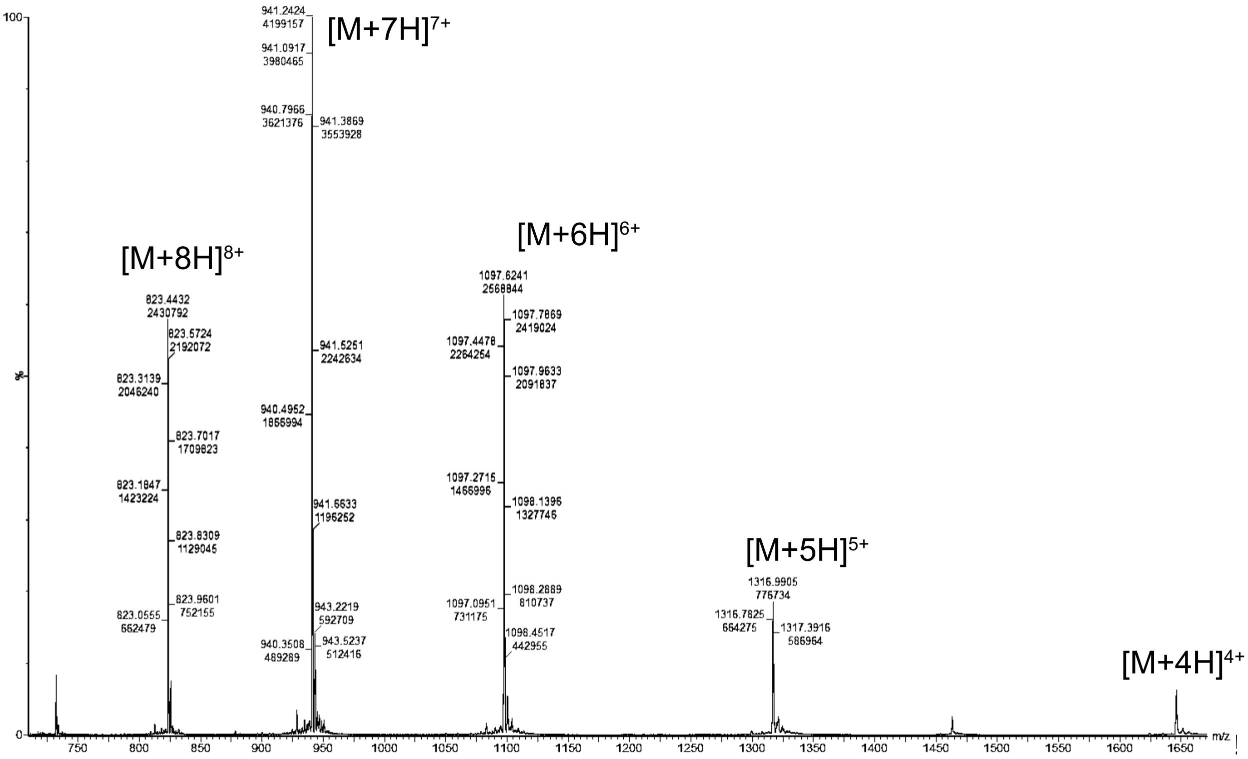

Expansion mass spectrum of trimer 2AT-L.

Calculated $[M+H]^+$ of trimer 2AT-L: 6577.34

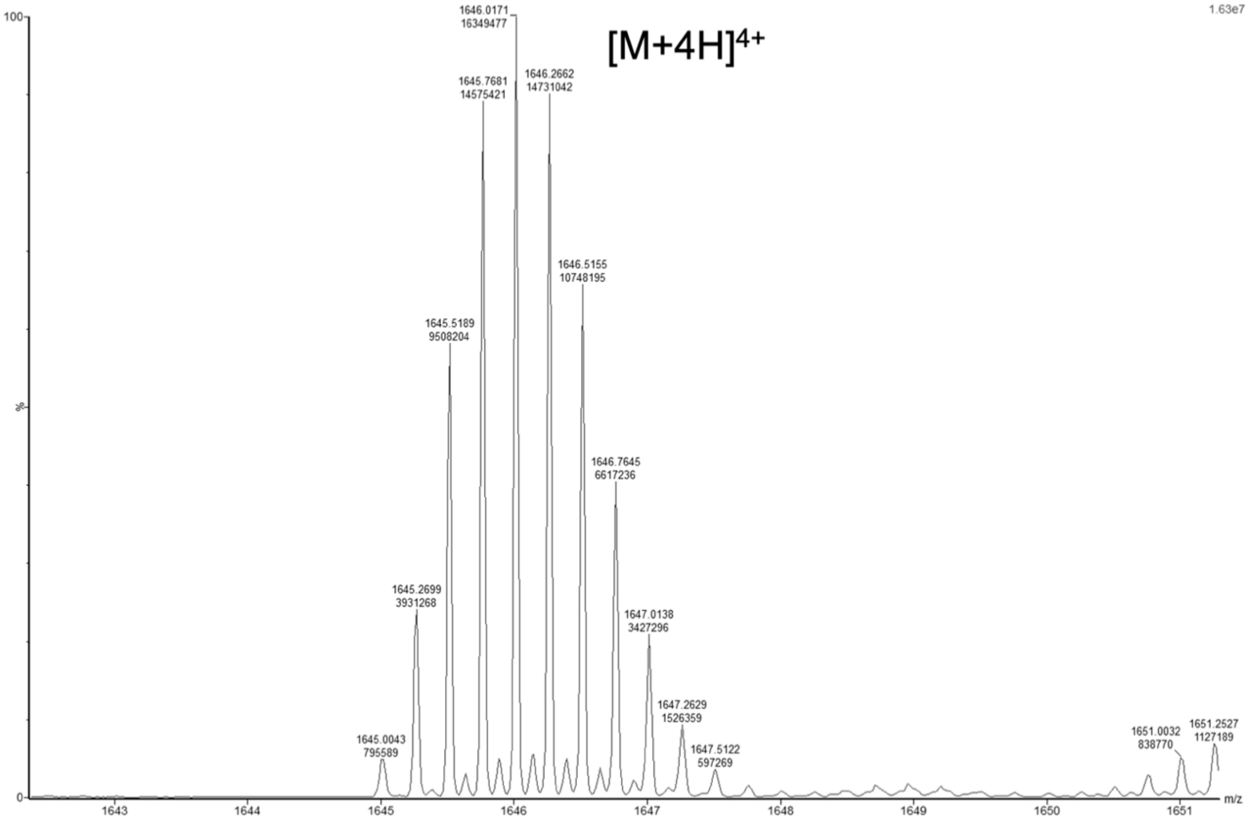

Deconvoluted mass spectrum of trimer 2AT-L.

Exact mass of trimer 2AT-L: 6576.34

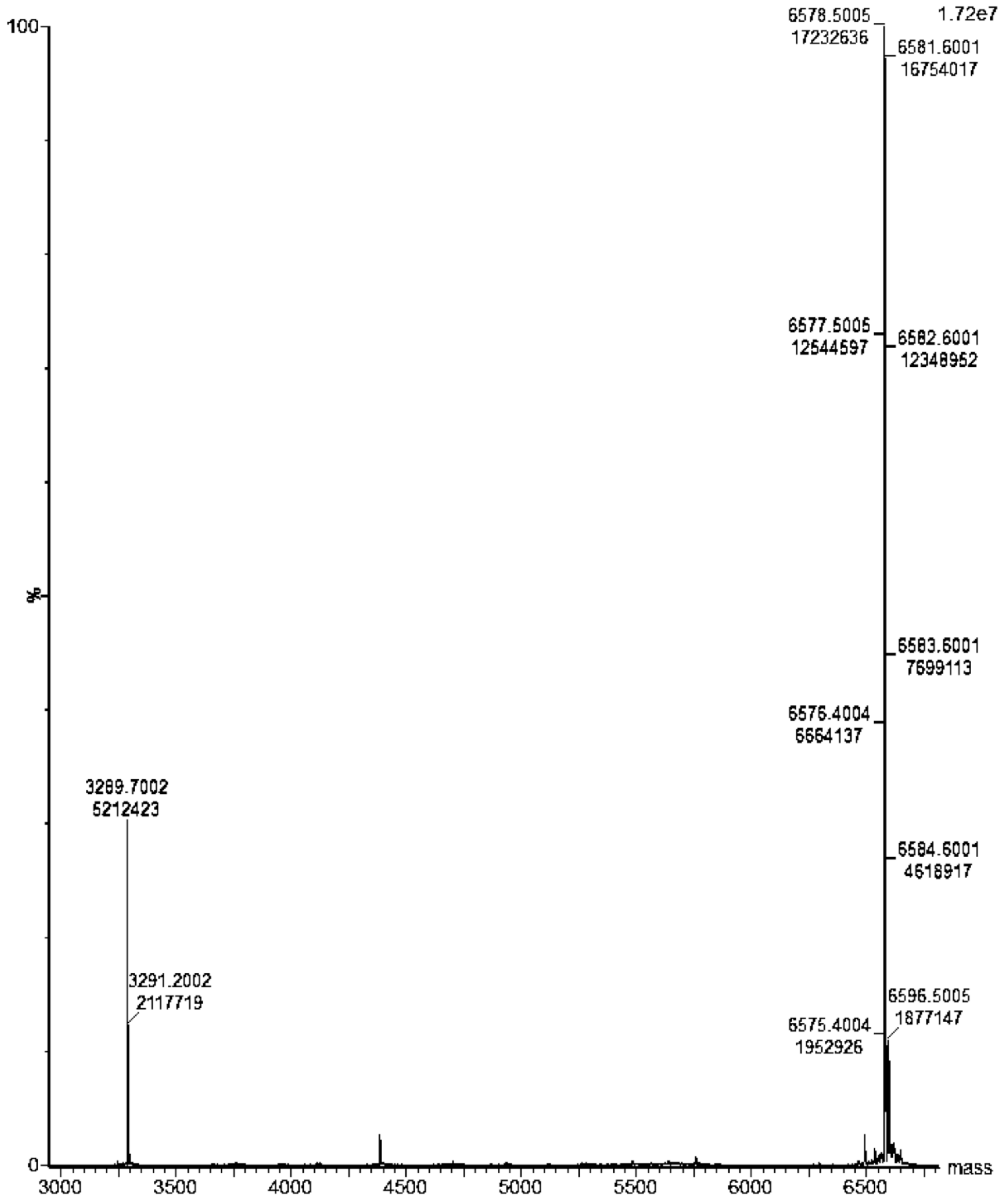

Deconvoluted mass spectrum of trimer 2AT-L.

Exact mass of trimer 2AT-L: 6576.34

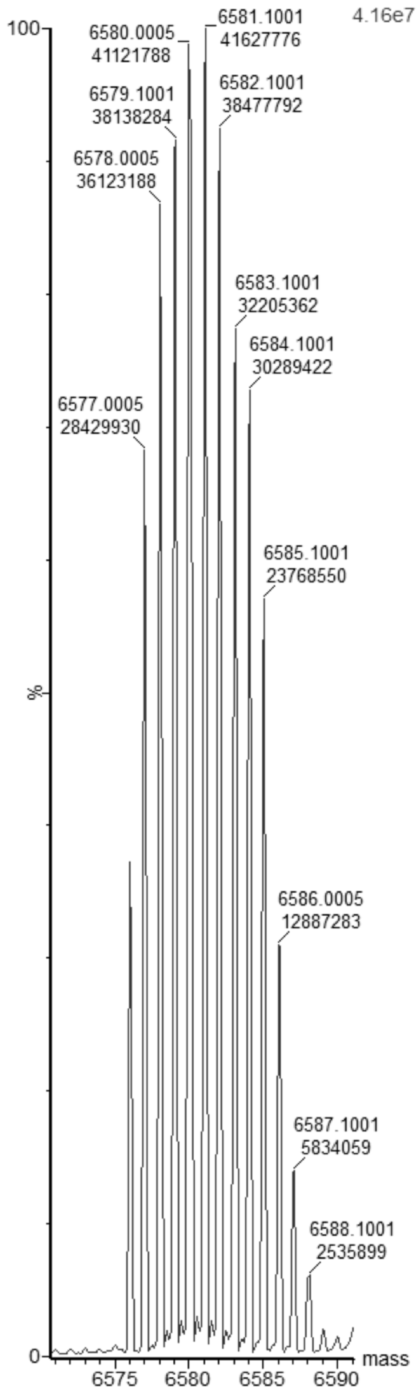

Characterization of trimer 2AT-L-sCy3

Analytical HPLC trace

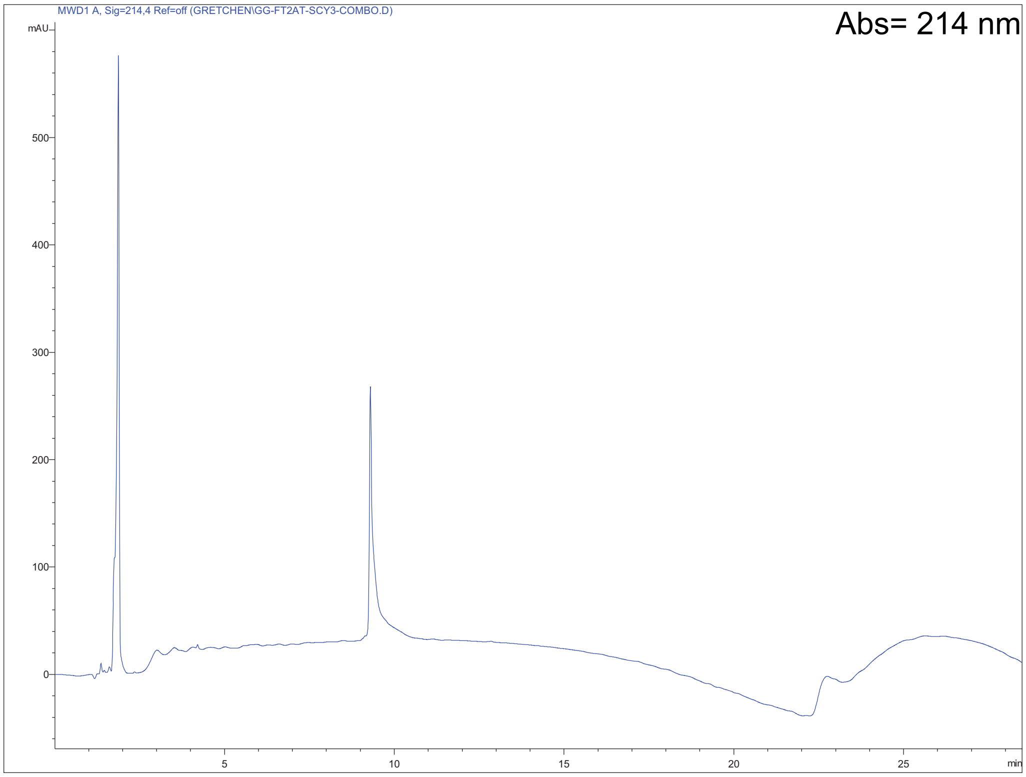

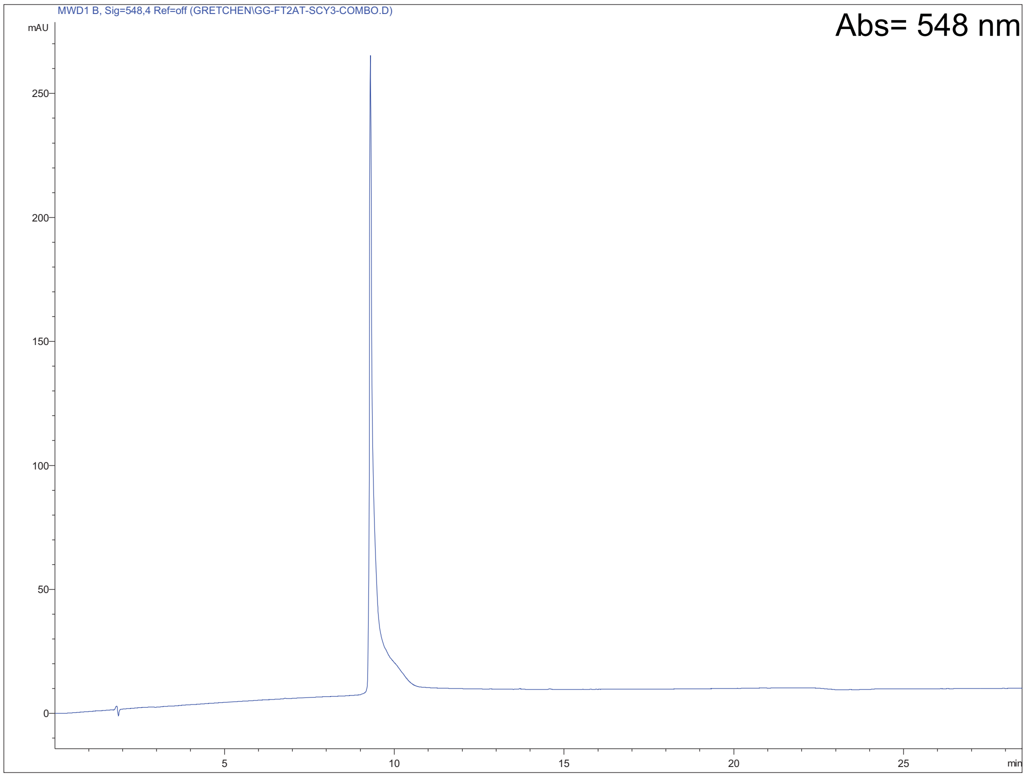

Mass spectrum of trimer 2AT-L-sCy3.

Calculated $[M+H]^+$ of trimer 2AT-L-sCy3: 7175.52

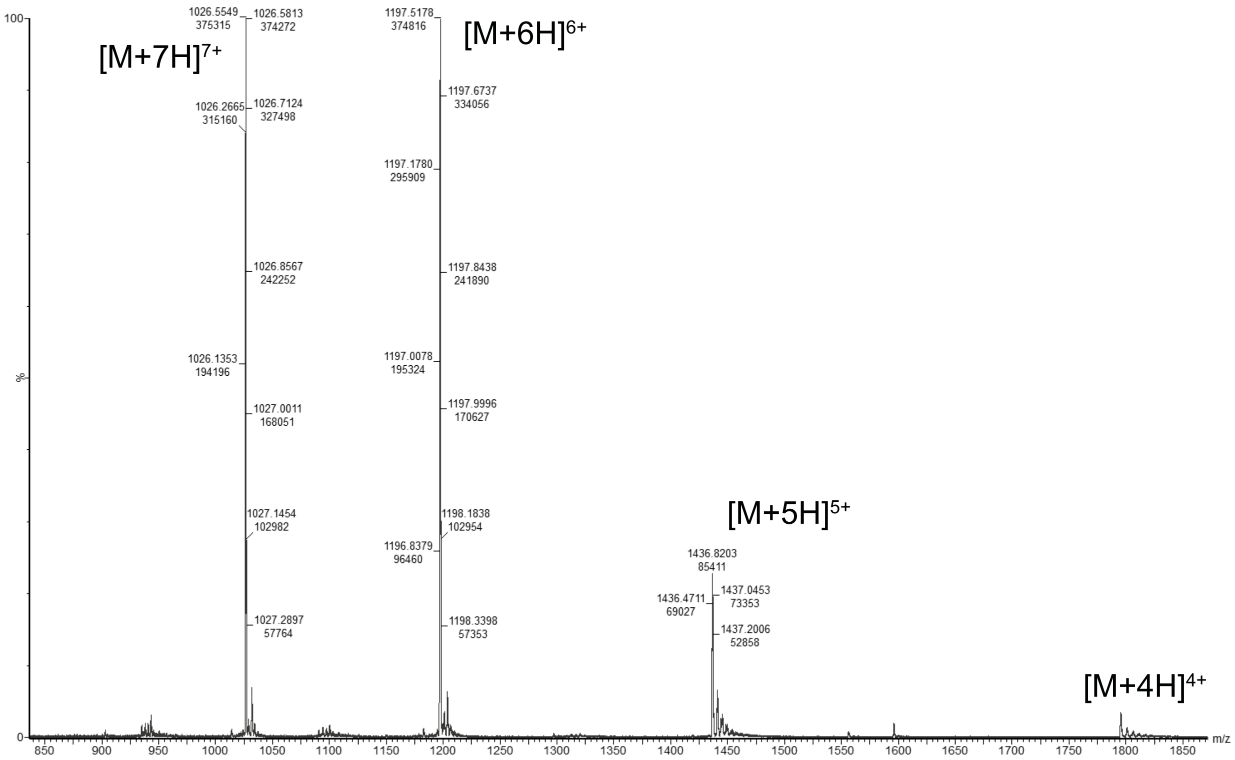

Expansion mass spectrum of trimer 2AT-L-sCy3.

Calculated $[M+H]^+$ of trimer 2AT-L-sCy3: 7175.52

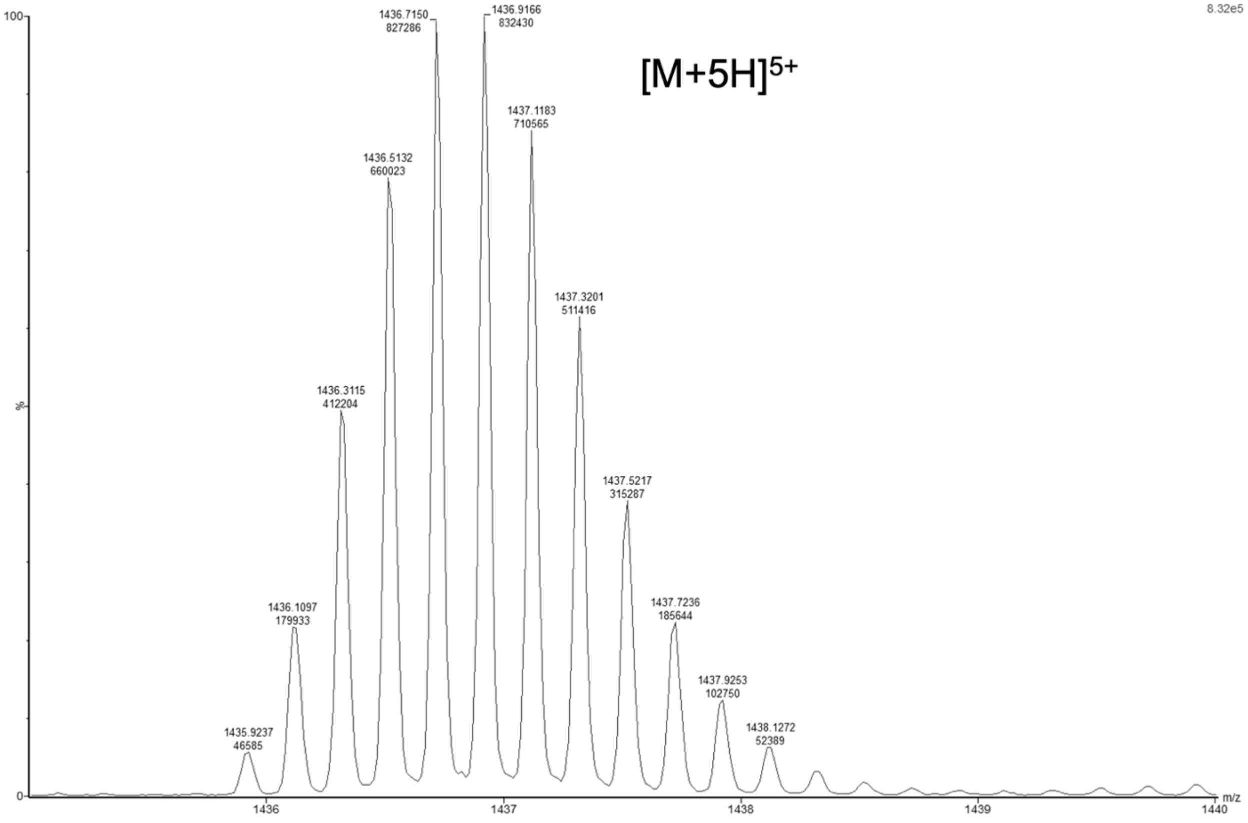

Deconvoluted mass spectrum of trimer 2AT-L-sCy3.

Exact mass of trimer 2AT-L-sCy3: 7174.52

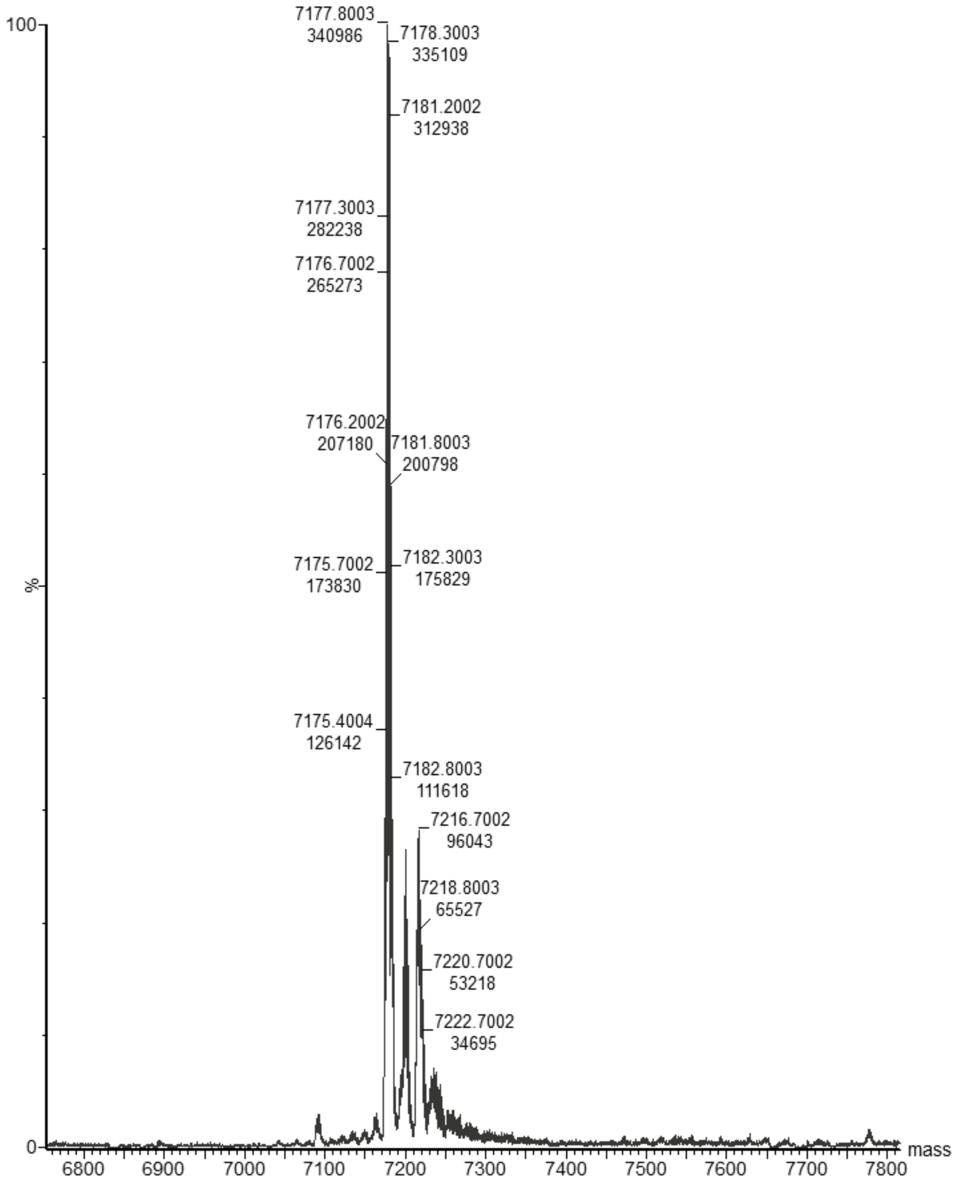

Deconvoluted mass spectrum of trimer 2AT-L-sCy3.

Exact mass of trimer 2AT-L-sCy3: 7174.52

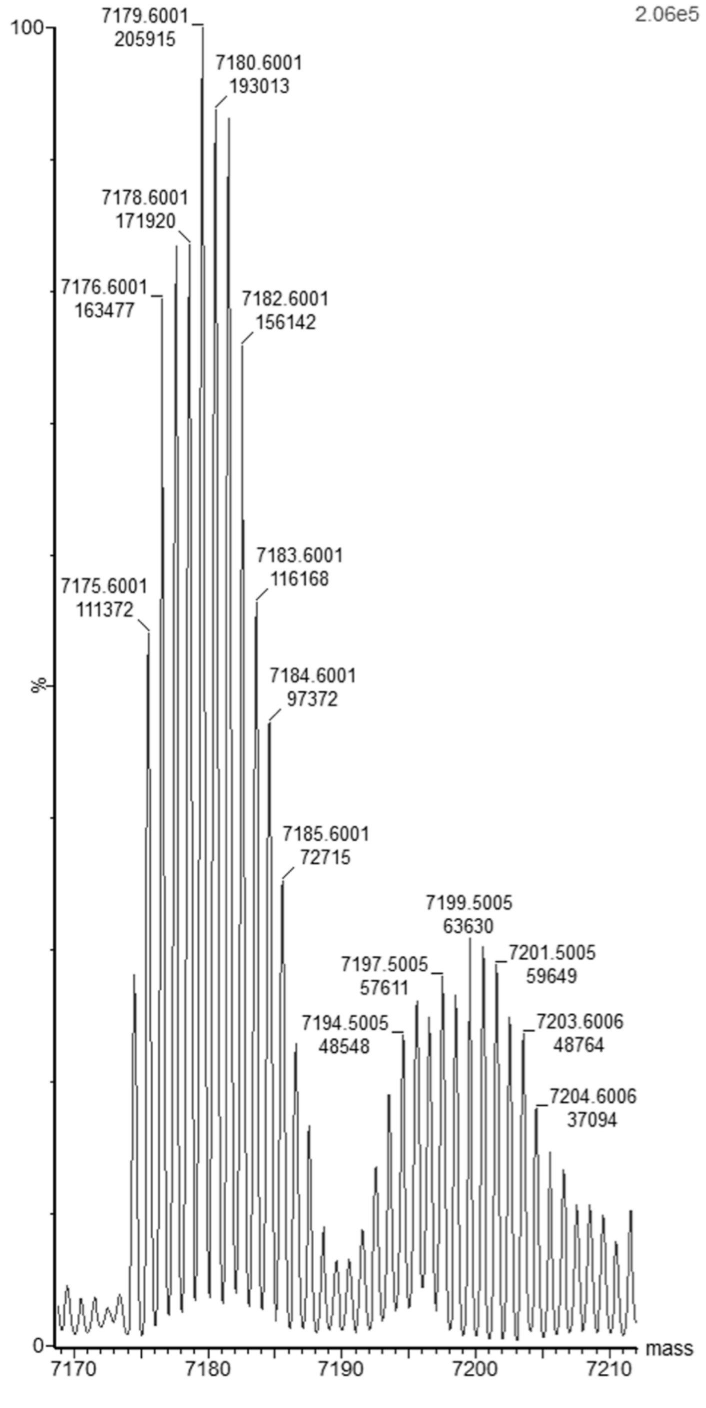

Characterization of trimer 2AT-L-sCy5

Analytical HPLC trace

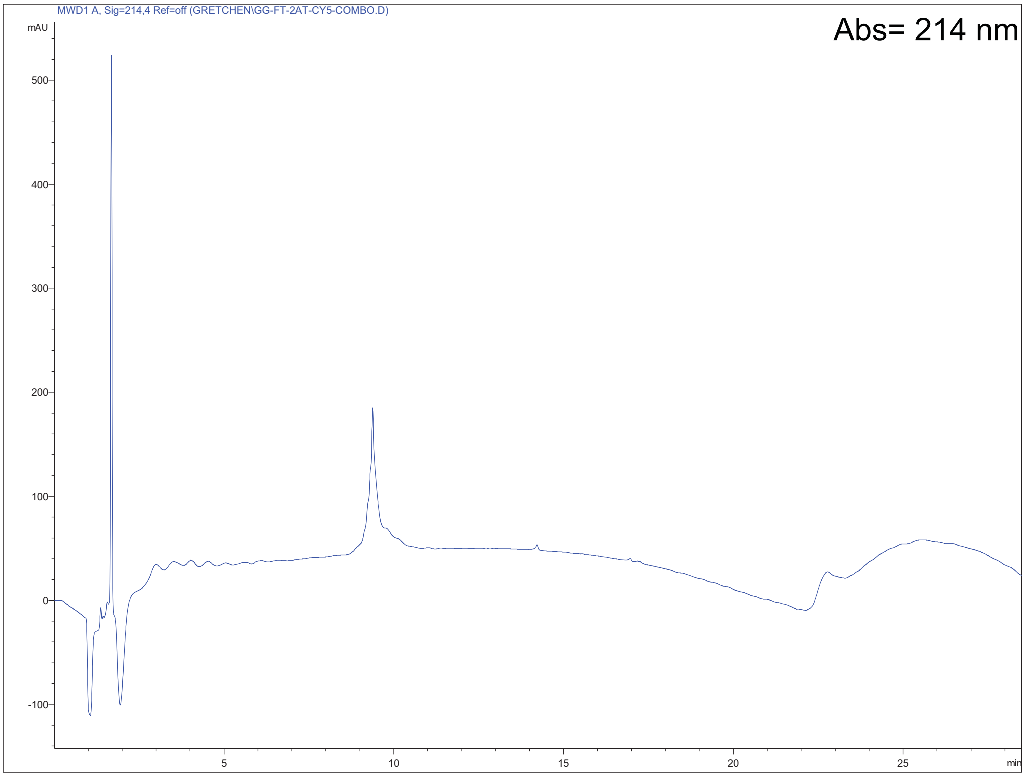

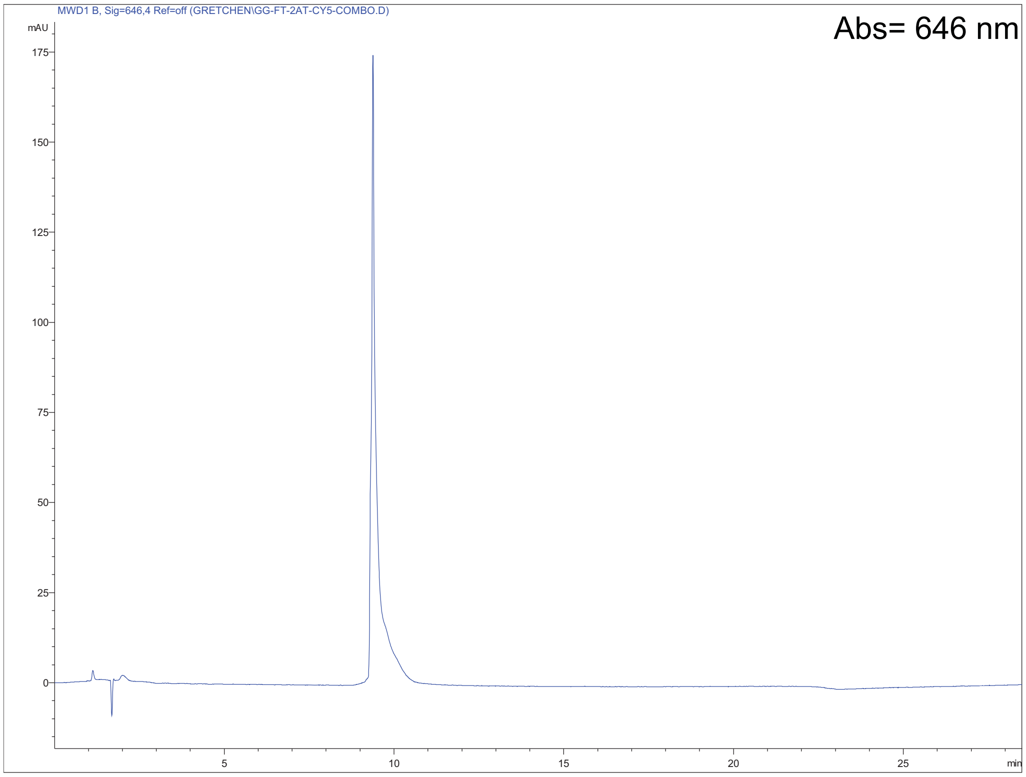

Mass spectrum of trimer 2AT-L-sCy5.

Calculated $[M+H]^+$ of trimer 2AT-L-sCy5: 7201.54

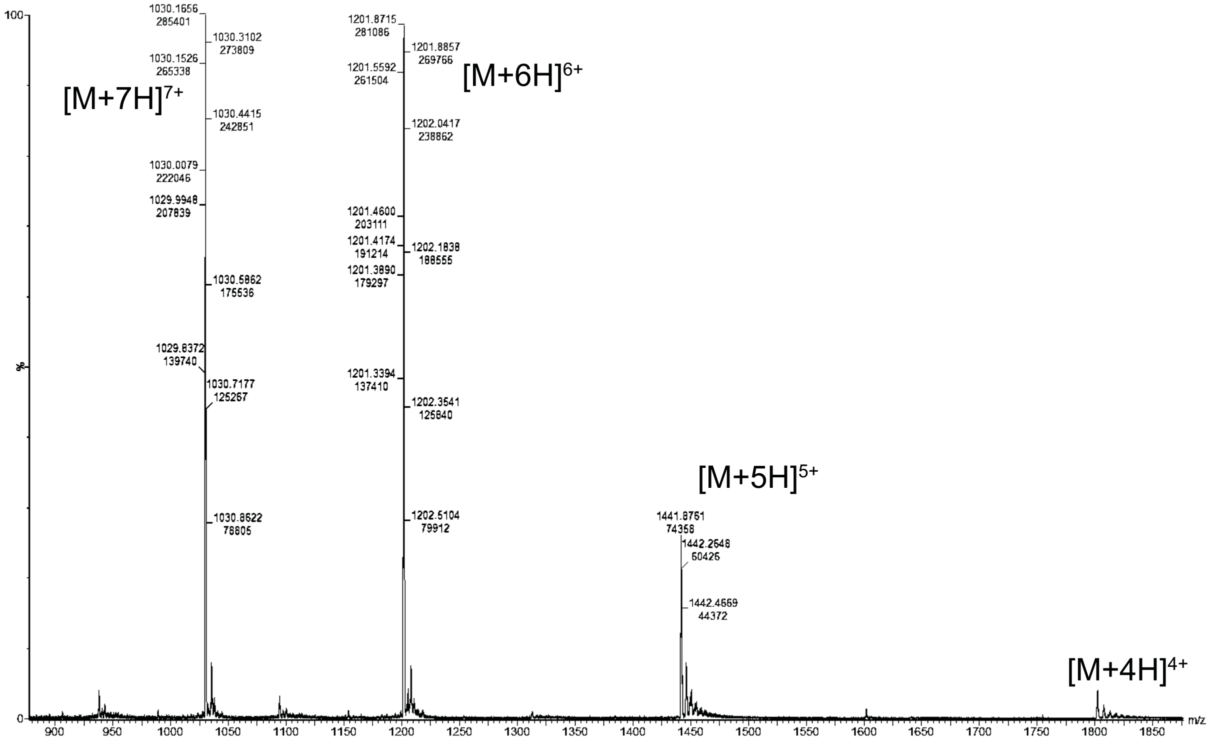

Mass spectrum of trimer 2AT-L-sCy5.

Calculated $[M+H]^+$ of trimer 2AT-L-sCy5: 7201.54

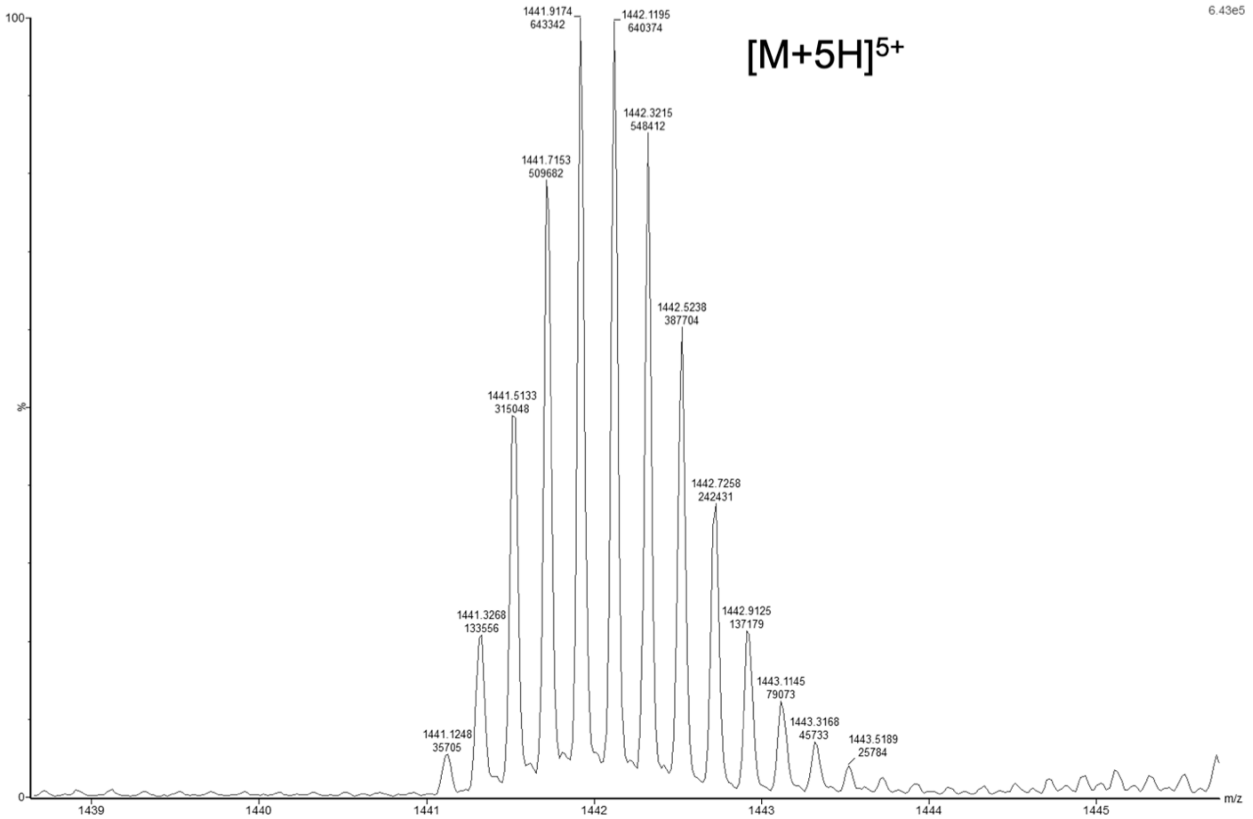

Deconvoluted mass spectrum of trimer 2AT-L-sCy5.

Exact mass of trimer 2AT-L-sCy5: 7200.53

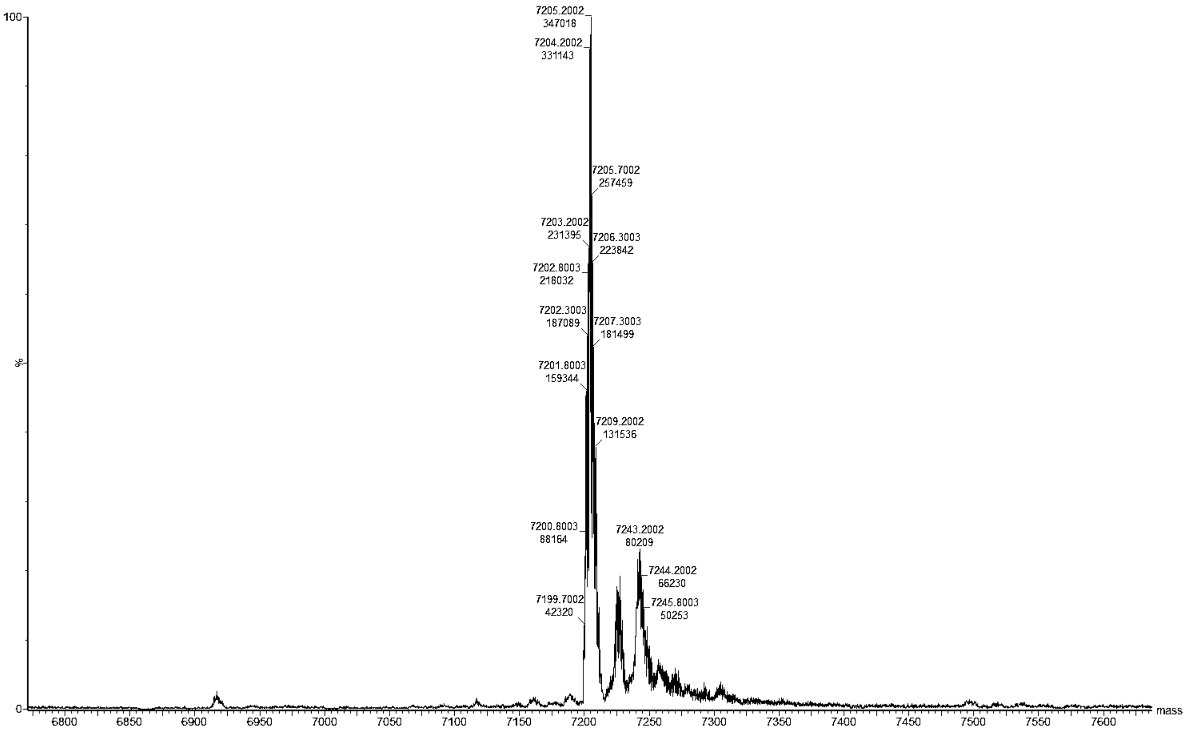

Deconvoluted mass spectrum of trimer 2AT-L-sCy5.

Calculated mass of trimer 2AT-L-sCy5: 7200.53

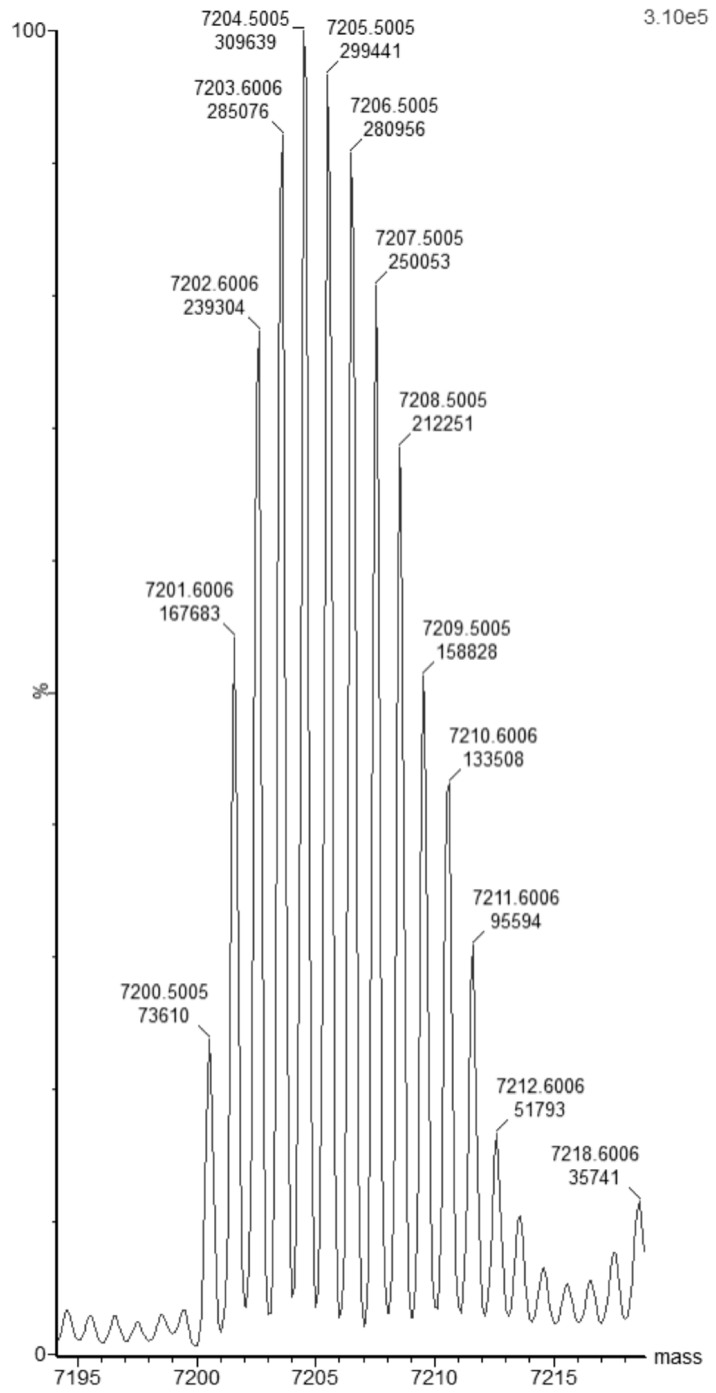

Supplement: Supplementary file 1 — cn2c00313_si_001.pdf [file cn2c00313_si_001.pdf]
